# A metabolic shift toward glycolysis enables cancer cells to maintain survival upon concomitant glutamine deprivation and V-ATPase inhibition

**DOI:** 10.3389/fnut.2023.1124678

**Published:** 2023-05-15

**Authors:** Florian Lengauer, Franz Geisslinger, Antje Gabriel, Karin von Schwarzenberg, Angelika M. Vollmar, Karin Bartel

**Affiliations:** Department of Pharmacy, Pharmaceutical Biology, Ludwig-Maximilians University, Munich, Germany

**Keywords:** cancer, V-ATPase, glycolysis, glutamine, cancer metabolism

## Abstract

It is widely known that most cancer cells display an increased reliance on glutaminolysis to sustain proliferation and survival. Combining glutamine deprivation with additional anti-cancer therapies is an intensively investigated approach to increase therapeutic effectiveness. In this study, we examined a combination of glutamine deprivation by starvation or pharmacological tools, with the anti-cancer agent archazolid, an inhibitor of the lysosomal V-ATPase. We show that glutamine deprivation leads to lysosomal acidification and induction of pro-survival autophagy, which could be prevented by archazolid. Surprisingly, a combination of glutamine deprivation with archazolid did not lead to synergistic induction of cell death or reduction in proliferation. Investigating the underlying mechanisms revealed elevated expression and activity of amino acid transporters SLC1A5, SLC38A1 upon starvation, whereas archazolid had no additional effect. Furthermore, we found that the export of lysosomal glutamine derived from exogenous sources plays no role in the phenotype as knock-down of SLC38A7, the lysosomal glutamine exporter, could not increase V-ATPase inhibition-induced cell death or reduce proliferation. Analysis of the cellular metabolic phenotype revealed that glutamine deprivation led to a significant increase in glycolytic activity, indicated by an elevated glycolytic capacity and reserve, when V-ATPase function was inhibited concomitantly. This was confirmed by increased glutamine uptake, augmented lactate production, and an increase in hexokinase activity. Our study, therefore, provides evidence, that glutamine deprivation induces autophagy, which can be prevented by simultaneous inhibition of V-ATPase function. However, this does not lead to a therapeutic benefit, as cells are able to circumvent cell death and growth inhibition by a metabolic shift toward glycolysis.

## 1. Introduction

Cancer is still a major of death cause worldwide and limited therapeutic options, severe therapy side effects, and poor clinical outcomes clearly depict the urgent need to develop novel strategies for this disease (1, 2). One important recently emerged hallmark of cancer is metabolic reprogramming. Otto Warburg and his co-workers showed early on that cancer cells produce lactic acid from glucose even under non-hypoxic conditions, since then known as the Warburg effect (3). Nowadays it is evident, that cancer cells undergo extensive metabolic reprogramming to meet their increased energetic demands. They alter cellular pathways to generate energy, produce co-factors for anabolic reactions, and build metabolic precursors, while several generated metabolites also exert a signaling function promoting tumor growth and progression. This extends not only to glycolysis, but also to glutamine, serine, methionine, arginine, and lipid metabolism (4).

A challenge when developing strategies is to decide which metabolic adaptation is best to address. A promising target in that regard is glutamine metabolism, or ‘glutaminolysis’. “Glutamine addiction,” i.e., excessive consumption of glutamine from human plasma or cell culture media in amounts beyond those necessary for protein synthesis, is exceedingly common across different tumor entities (5). Glutamine is imported into the cells by specialized amino acid transporters, such as SLC38A1 and SLC1A5 and transported into mitochondria mainly for energy generation. Glutamine is hydrolyzed to glutamate by glutaminases and can be further metabolized by glutamine dehydrogenase (GLUD) or glutamic-oxaloacetic transaminase (GOT) to α-ketoglutarate (αKG), which feeds into the TCA cycle to provide energy and building blocks (6). The metabolic pathways glutamine feeds into are versatile and at the same time essential for tumor growth and survival. Therefore, targeting glutamine metabolism represents a promising anti-cancer approach (7).

In recent research, the lysosomes turned out as master regulators of cellular nutrient sensing and adaptation to metabolic stress. Cellular glucose and amino acid levels are sensed in lysosomes by the LYNUS (lysosomal nutrient sensing) machinery. This multi-protein complex resides on the lysosomal surface and includes the V-ATPase, a lysosomal proton pump responsible for luminal acidification, and mTORC1, a master regulator of metabolic adaptation processes, which is tethered to the V-ATPase by several adapter proteins (8). Upon starvation, mTORC1 is inactivated, leading to the induction of lysosomal biogenesis and autophagy to maintain cellular energy generation (9). Recently, the V-ATPase has also been implicated in cellular metabolism and adaptation to starvation conditions by mTORC1 and AMPK signaling (10). There is also initial evidence that targeting V-ATPase influences glutamine dependence of cancer cells (11). Additionally, luminal acidification of lysosomes, facilitated by the V-ATPase, is a decisive prerequisite for fusion with autophagosomes and subsequent cargo degradation. V-ATPase inhibition is hence an effective way to inhibit autophagy (12). Given the role of V-ATPase nutrient sensing and autophagy, our working hypothesis was that inhibition of autophagy by V-ATPase inhibition, concomitantly with glutamine-deprivation, leads to increased growth-inhibition and apoptosis, especially in glutamine-dependent cancer cells. This strategy may be a novel therapeutic option and thus underlying cellular signaling processes warrant thorough examination.

## 2. Methods

### 2.1. Compounds and cell culture

HCT-15, BxPC3, Panc03.27, Panc10.05 and HT-29 cells were obtained from ATCC. STR analysis and testing for mycoplasma contamination were performed regularly. Cells were grown in DMEM supplemented with 10% FCS (PAN-Biotech GmbH, Aidenbach, Germany) and cultured under constant humidity at 37°C, 5% CO_2_. Archazolid A was kindly provided by Rolf Müller (Saarland University, Saarbrücken, Germany). BPTES, CB-839, Oligomycin, and 2-Deoxyglucose were purchased from Merck Millipore kGaA (Darmstadt, Germany). Compounds were dissolved in DMSO.

### 2.2. Flow cytometry

Cells were analyzed by flow cytometry using a FACSCanto™II, FACSDiva™, and FlowJo™10.8.1 (Becton Dickinson GmbH, Heidelberg, Germany). Apoptosis was assessed as described previously (13) by propidium iodide (50 μg/mL) staining after 48 h, after harvesting on ice and permeabilization in fluorochrome solution (0.1% (w/v) sodium citrate, 0.1% (v/v) Triton X-100, PBS) (Carl Roth GmbH, Karlsruhe, Germany). Lysosomes were stained after 1 h treatment, with 100 nM LysoTracker™ Green or 1 μM LysoSensor™ Green for 30 min, at 37°C. Transferrin-AlexaFluor™488 (5 μg/mL, 30 min) uptake was analyzed after 1 h treatment (Dyes from: Thermo Fisher Scientific Inc., Waltham, MA, USA). For analysis of glucose uptake, cells were loaded with 100 μM 2-NBDG (Bio-gems, Westlake Village, CA, USA) for 30 min prior to harvesting.

### 2.3. Confocal microscopy

Cells were seeded into IbidiTreat 8-well μ-slides (Ibidi GmbH, Martinsried, Germany). After 24 h treatment, cells were fixed in 4% Paraformaldehyde (10 min), and permeabilized with 0.5% Triton-X (10 min) (Carl Roth GmbH, Karlsruhe, Germany). Unspecific binding was blocked with 5% BSA (1 h), followed by addition of primary antibodies (Supplementary Table 4) (2 h) and secondary (1 h) (Supplementary Table 5) together with Hoechst 33342 (Merck Millipore kGaA, Darmstadt, Germany) and Rhodamine/Phalloidin Red (#R-415; Thermo Fisher Scientific Inc., Waltham, MA, USA). For semi-quantitative pH measurements, cells were loaded with FITC-Dextran (20 kDa, 200 μg/mL, 24 h), and treated for 1 h, followed by Hoechst 33342 staining. Images were acquired utilizing a Leica TCS SP8, LasX software (Leica Microsystems GmbH, Wetzlar, Germany) and ImageJ (National Institutes of Health, Bethesda, MD, USA).

### 2.4. Western Blot

After 24 h treatment, cells were harvested and lysed in detergent-containing buffer (50 mM Tris/HCl, 150 mM NaCl, 1% Non-idet NP-40, 0.25% Sodium deoxycholate, 0.10% SDS, 0.5 mM PMSF, 2 mM Na_3_VO_4_ in deionized water; pH 7.5 and complete™ (Roche Holding AG, Basel, Switzerland). Protein concentrations were determined as described previously (14). Sample buffer (3.125 M Tris-HCl (pH 6.8), 50% glycerol, 5% SDS, 2% DTT, 0.025% Pyronin Y in deionized water) was added, and equal amounts of protein were separated by SDS-PAGE (100 V/21 min, 200 V/40 min). Loading was determined by stain-free technology (15). Proteins were transferred to a 0.2 μm PVDF membrane (Hybond-ECL™, Amersham Bioscience, Freiburg, Germany) by tank blotting (100 V/1.5 h/4°C). Unspecific binding was blocked with 5% BSA (PBS, 0.5% Tween-20^®^, 2 h), followed by addition of primary antibodies (Supplementary Table 4) (24 h), and appropriate secondary antibodies conjugated to HRP (1 h) (Supplementary Table 5). Proteins were detected using ECL solution (100 mM Tris-HCl, 2.5 mM Luminol, 1 mM Coumaric acid, 17 mM H_2_O_2_ in deionized water), and analyzed using a ChemiDoc™ Touch Imaging System, and Image Lab™ (Bio-Rad Laboratories Inc., Hercules, CA, USA).

### 2.5. Proliferation

Cells were stained with crystal violet (0.5% crystal violet (w/v), 50% methanol (v/v); 10 min) after 72 h treatment. Crystal violet was redissolved with 50% ethanol (v/v), 50% 0.1 M sodium citrat (w/v) and absorption was measured at 550 nm using a Tecan SpectraFluor-Plus™ (Tecan AG, Männedorf, Switzerland). Half-maximal inhibitory concentrations (IC_50_-values) were calculated by non-linear regression using GraphPad Prism 8.2.1 software (GraphPad Software Inc., San Diego, CA, USA). For knockdown experiments, cells were transfected 24 h prior to treatment with non-targeting control siRNA or siRNA targeting SLC38A7 using DharmaFECT™ transfection reagent (Dharmacon™, GE Healthcare, Lafayette, LA, USA) according to manufacturer’s protocol.

### 2.6. Quantitative real-time PCR analysis

mRNA was isolated using the RNeasy^®^ Mini Kit (250) (QIAGEN, Hilden, Germany) as described by the manufacturer, and concentration was determined using a Nanodrop^®^ Spectrophotometer (PEQLAB Biotechnologie, Erlangen, Germany). Reverse transcription was performed with the High-Capacity cDNA Reverse Transcription Kit (Thermo Fisher Scientific Inc., Waltham, MA, USA) according to the manufacturer’s instructions. 100 ng of cDNA (2 μL), 6.25 μL PowerUp™ SYBR^®^ Green Master Mix (Thermo Fisher Scientific Inc., Waltham, MA, USA), 3.25 μL RNase-free water (Thermo Fisher Scientific Inc., Waltham, MA, USA) and 0.025 mol of each primer (Supplementary Table 6), Metabion international AG, Planegg, Germany) were used for qPCR reaction using a MicroAmp^®^ Fast Optical 96-Well Reaction Plate in a QuantStudio™ 3 Real-Time PCR System (Thermo Fisher Scientific Inc., Waltham, MA, USA). Quantification was performed using the ΔΔC_*T*_ method (16), actin served as housekeeping gene.

### 2.7. Glutamine uptake assay

L-[3 H]glutamine (9.25 MBq/mL) was used at 2,000 dpm/nmol with unlabeled L-glutamine adjusted to 100 μM final concentration. Stock solution was made of 991 μL 10 mM unlabeled L-glutamine and 9 μL L-[3 H]glutamine. After treatment, growth medium was removed and cells were washed twice with 1.5 mL pre-warmed Hank’s balanced salt solution (HBSS) (1.26 mM CaCl_2_, 5.56 mM D-glucose, 5.33 mM KCl, 0.49 mM MgCl_2_ × 6 H_2_O, 0.44 mM KH_2_PO_4_, 0.41 mM MgSO_4_ × 7 H_2_O, 0.34 mM Na_2_HPO_4_, 137.9 mM NaCl, 4.17 mM NaHCO_3_ in deionized water). Subsequently, L-[3 H]glutamine was added in 1.5 mL HBSS (100 μM) for 20 min at 37°C. After washing, supernatant was discarded and cells were lysed using 300 μL lysis buffer containing 0.2 M NaOH and 0.1% SDS in PBS for 1 h). To normalize transport rates, 40 μL of cell lysate were neutralized with 0.2 N HCl and protein-concentration was measured as described previously (14). The remaining lysate was added to 15 mL scintillation cocktail prepared in scintillation tubes and intracellular radioactive glutamine was quantified using a LS6500 liquid scintillation counter (Beckman Coulter, Brea, CA, USA). Values were measured in counts per minute (CPM) and normalized to cell-derived protein content giving transport rates in cpm/μg protein.

### 2.8. Metabolic stress test

After treatment for 24 h on a sensor plate, media reservoirs were filled with basic measuring media (DMEM, supplemented with 4.5 g/L glucose, pH 7.2) supplemented with 584 mg/L [Gln(+)] or 116.8 mg/L [Gln(low)] glutamine and 10% FCS. Medium was replaced with respective measuring media, the plate was sealed and loaded into the CYRIS^®^ flox (INCYTON^®^ GmbH, Planegg, Germany) (17). 1 μM Oligomycin, and 0.5 mM 2-Deoxyglucose (2-DG) were applied sequentially (4 cycles of 12 min each). The obtained raw data was normalized to cell density (crystal violet staining).

### 2.9. SeaHorse™ measurements

XFe96 microplates (Agilent Technologies Inc., Santa Clara, CA, USA) were pre-coated with Cell-Tak™ cell and tissue adhesive solution (22.4 μg/mL, 25 μL/well, 20 min) (Corning Inc., New York, NY, USA), washed, and cells were seeded. The Seahorse XFe96 sensor cartridge was hydrated, extracellular flux analysis and glycolysis stress test were performed as indicated by the manufacturer on a Seahorse the XFe96 Analyzer. Data were analyzed with Wave 2.6.1 software (Agilent Technologies Inc., Santa Clara, CA, USA). For data evaluation, ECAR (extracellular acidification rate) was normalized to cell number, assessed via Hoechst 33342 staining measured by BioTek Cytation Cell Imaging Reader (Agilent Technologies Inc., Santa Clara, CA, USA).

### 2.10. Lactate production

Lactate production was assessed using Lactate-Glo™ assay kit (Promega) as described by the manufacturer (#J5021; Promega Inc., Fitchburg, WI, USA). Reagents were prepared as indicted in 3A in the manufacturers protocol. Cells were treated as indicated for 24 h, samples prepared as described in 4A by manufacturer and diluted 1:50 with PBS. After mixing and incubating, luminescence was detected with an Orion II microplate luminometer (Berthold Detection Systems GmbH, Pforzheim, Germany). Luminescence signal correlates with presence of lactate in the sample.

### 2.11. Hexokinase activity assay

The enzyme activity of Hexokinase was assessed according to the manufacturer’s protocol by Hexokinase activity assay kit (#ab136957; Abcam Inc., Cambridge, UK).

### 2.12. Statistical analysis

Data are presented as mean ± SEM of three independent experiments and statistical differences were assessed with an ordinary Two-way ANOVA/Tukey’s multiple comparisons test using GraphPad Prism (GraphPad Software Inc., San Diego, CA, USA) unless stated otherwise.

## 3. Results

**FIGURE 1:**
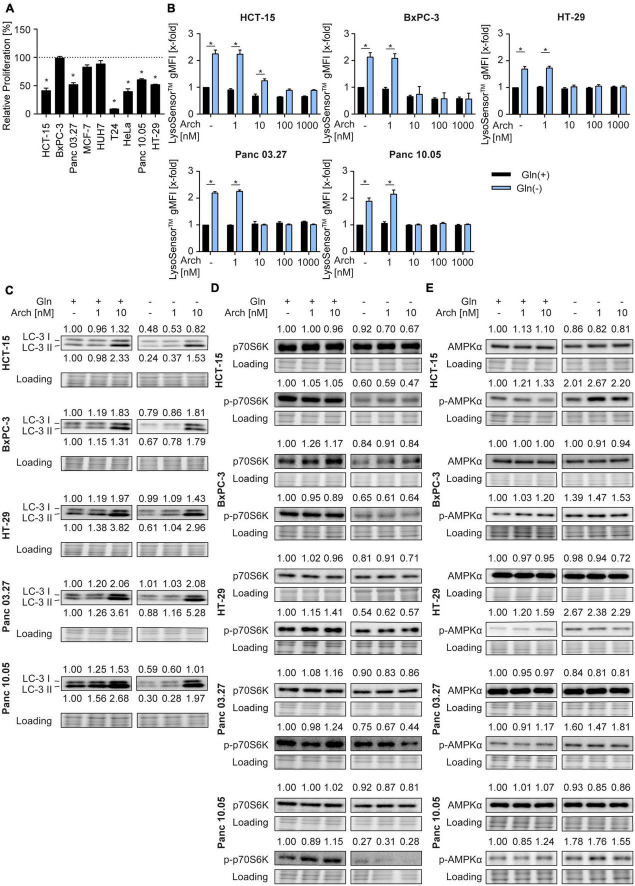
Lysosomal characteristics upon glutamine starvation and V-ATPase inhibition. **(A)** Proliferation was determined by crystal violet staining. Cells were treated as indicated for 72 h. Bar graphs display mean ± SEM (n = 3), One-way ANOVA followed by Tukey’s multiple comparison test. *p < 0.05. **(B)** Cells were treated as indicated for 24 h followed by LysoSensor™ Green FM staining. LysoSensor™ Green FM intensity was assessed by flow cytometry analysis and displayed as geometric mean of fluorescent intensity (gMFI). **(C–E)** LC-3 I and II **(C)**, p70S6K and phosphorylated p70S6K **(D)**, AMPKα and phosphorylated AMPKα **(E)** protein levels were analyzed by western blotting after 24 h of treatment as indicated. Quantification is indicated above (and below for LC3-II) the respective bands. One representative Western Blot is shown. **(B–E)** Results are mean ± SEM (n = 3), Two-way ANOVA followed by Tukey’s multiple comparison test. *p < 0.05.

To investigate the potential of the V-ATPase inhibition combined with glutamine deprivation in cancer cells, we first determined glutamine dependency in different cancer cell lines, originating form colorectal carcinoma (HCT-15, HT-29), pancreatic cancer (BxPC3, Panc03.27, Panc10.05), breast cancer (MCF-7), hepatocellular carcinoma (HUH-7), bladder cancer (T24), and cervical cancer (HeLa). Therefore, we analyzed proliferation capacity of these cells in the presence or absence of glutamine. Glutamine deprivation led to a reduction in proliferative capacity in all tested cells, however, to different extents (Figure 1A). As a study model, we focused on colorectal cancer and pancreatic cancer lines for two main reasons: (a) glutamine dependency has been reported in these tumor entities also in clinical settings (18, 19) and (b) they showed medium (about 40-60%) reduction in proliferation, which is a feasible amount for implementation of a potential combination therapy strategy. Since lysosomes are the cellular centers for nutrient sensing, we first assessed the impact of glutamine deprivation on lysosomal characteristics. While lysosomal volume was unchanged as indicated by LysoTracker™ Green staining (Supplementary Figure 1A), lysosomes were more acidic upon glutamine-deprivation indicated by semi-quantitative analysis of LysoSensor™ Green fluorescence (Figure 1B). This was further confirmed by pH-sensitive quenching of FITC-dextran fluorescence (Supplementary Figure 1B). This phenomenon could be reversed by simultaneous treatment with the V-ATPase inhibitor archazolid (Figure 1B and Supplementary Figure 1B).

As glutamine starvation has been reported to induce autophagy as a survival mechanism (20) and that V-ATPase inhibition blocks autophagy (16), we analyzed autophagy. In our model, glutamine deprivation led to a decrease in LC3-II protein levels, indicating autophagy induction. As expected, V-ATPase inhibition could reverse this effect and block autophagic flux (Figure 1C). Of note, p62 levels remained unaffected (Supplementary Figure 1C). Analysis of phosphorylation of its downstream target p70S6K revealed decreased mTORC1 activity, evident by a reduction in p70S6K phosphorylation, which was also not further affected by archazolid co-treatment (Figure 1D). Furthermore, glutamine deprivation led to increased phosphorylation of the cellular energy sensor AMPK, which was not affected by V-ATPase inhibition (Figure 1E). Taken together, glutamine deprivation apparently induces changes in lysosomal properties and autophagic flux of cancer cells, which can be reversed by V-ATPase inhibition. These data support our hypothesis, that inhibiting V-ATPase in glutamine-deprived conditions could block cellular survival mechanisms and might be beneficial for cancer therapy.

**FIGURE 2:**
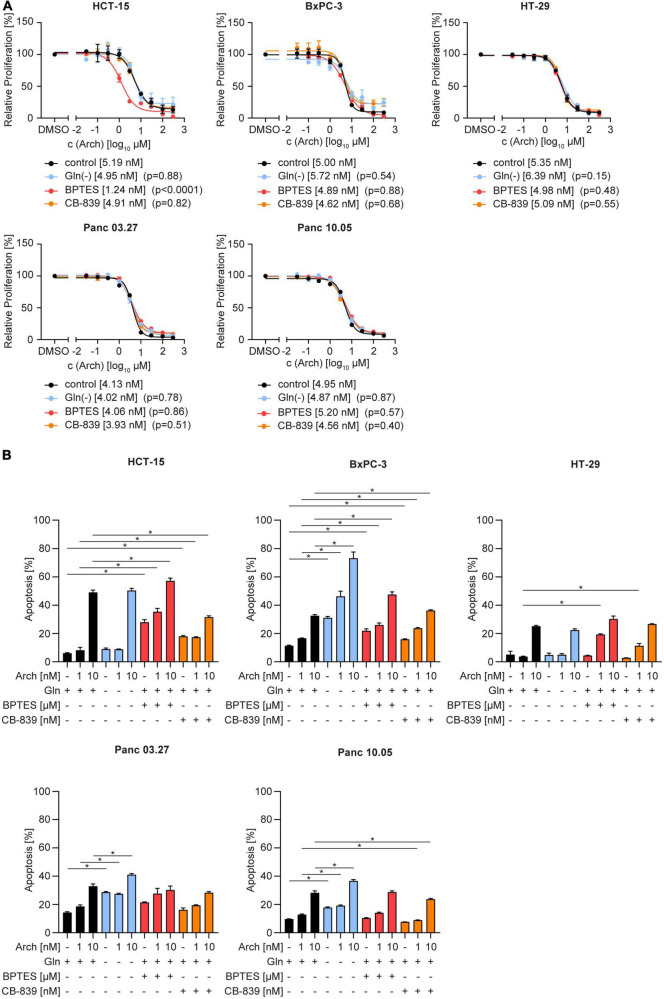
Proliferation and apoptosis upon concomitant starvation and V-ATPase inhibition. **(A)** Proliferation was determined by crystal violet staining. Cells were treated as indicated for 72 h. Data are mean ± SEM (n = 3). Statistical significance was analyzed using the comparison of fits function of GraphPad Prism 8, comparing logIC50 or logEC50values. **(B)** Apoptosis was assessed after 48 h by Nicoletti assay followed by flow cytometry analysis. Bar graphs display mean ± SEM (n = 3), Two-way ANOVA followed by Tukey’s multiple comparison test. *p < 0.05.

To test the therapeutic benefit, we analyzed proliferation and apoptosis upon glutamine deprivation by starvation or pharmacological glutaminase inhibition with BPTES or CB-839 together with V-ATPase inhibition. Using crystal violet staining, we found no difference in dose-sensitivity to archazolid in full-medium versus glutamine-deprived conditions. The same phenotype was observed upon glutaminase inhibition with CB-839 or BPTES (Figure 2A). Additionally, we did observe a moderate increase in apoptosis upon the combination, yet no synergistic effect as hypothesized (Figure 2B). Of note, we could observe a significant effect on proliferation and on apoptosis for a combination of BPTES with archazolid in HCT-15 cells (Figures 2A, B). However, BPTES is an inhibitor of the multidrug efflux transporter P-gp (Supplementary Figure 2), of which archazolid is a known substrate. Hence the additive effect might result from an increased intracellular drug concentration, rather than from glutaminase inhibition. Taken together, these findings indicate that contrary to our hypothesis, V-ATPase inhibition has no therapeutic benefit in glutamine-deprived cancer cells despite reversing lysosomal and autophagic effects of glutamine deprivation (Figure 1).

**FIGURE 3:**
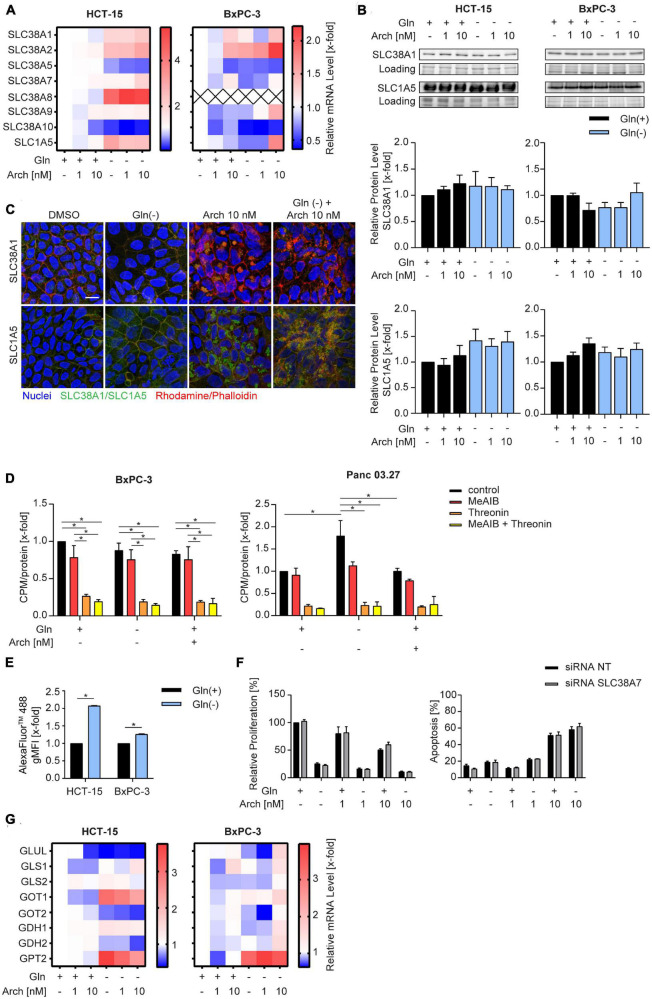
Influence of combination treatment on amino acid transporters. **(A)** mRNA levels were analyzed by qPCR after 24 h of treatment as indicated. Heatmaps display median values (n = 3). **(B)** SLC38A1 and SLC1A5 protein level in HCT-15 and BxPC-3 cells analyzed by western blotting after 24 h of treatment as indicated accompanied by quantification. One representative Western Blot is shown. **(C)** Cells were treated at the indicated concentrations for 24 h before staining with Hoechst 33,342 (nuclei, blue), Rhodamine/Phalloidin (actin, red) and SLC38A1 or SLC1A5, respectively (green) and analysis by confocal microscopy. Scale bar: 10 μM. One representative image is shown. **(D)** Uptake of L-[3H]-glutamine was measured in BxPC-3 (n = 3) and Panc 03.27 (n = 2) in the absence and presence of competing amino acids or analogues. Glutamine uptake was measured after 20 min and normalized to protein levels. **(E)** Cells were loaded with 200 μg/mL of FITC-Dextran 20kDa (green) for 24 h. Subsequently cells were treated at the indicated concentrations for 1 h before staining with Hoechst 33,342 (nuclei, blue) and analysis by confocal microscopy. Scale bar: 10 μM. One representative image is shown. Quantification of mean FITC intensity of five independent images per condition was performed by ImageJ software. **(F)** Proliferation was determined in HCT-15 cells by crystal violet staining. Cells were transfected 24 h prior to stimulation with non-targeting control siRNA or siRNA targeting SLC38A7 using DharmaFECT™ transfection reagent. Subsequently cells were treated as indicated for 72 h. **(G)** mRNA levels were analyzed by qPCR after 24 h of treatment as indicated. Heatmaps display median values (n = 3). Bar graphs display mean ± SEM (n = 3), Two-way ANOVA followed by Tukey’s multiple comparison test. *p < 0.05.

We were then curious to understand why there was no beneficial therapeutic effect of V-ATPase inhibition. We hypothesized that the cancer cells might be able to circumvent proliferation inhibition or apoptosis, by metabolic reprogramming. Therefore, we analyzed solute carrier family transporters (SLC) which have broad substrate specificity, including all neutral amino acids, like glutamine and alanine. Most SLCs were transcriptionally upregulated in HCT-15 cells and downregulated in BxPC-3 cells upon glutamine deprivation, while V-ATPase inhibition had no additional effect (Figure 3A and Supplementary Table 1). Ubiquitously expressed transporters SLC38A1 and SLC1A5 (21) were modestly upregulated in HCT-15 cells on mRNA and protein level, but not in BxPC-3 cells (Figures 3A, B). Confocal microscopy showed, that glutamine deprivation has no effect on transporter localization, while archazolid treatment leads to an accumulation of transporters in intracellular vesicles (Figure 3C). To assess transporter activity, we pre-starved the cells and then monitored L-[^3^H]-glutamine uptake, which was unchanged in BxPC-3 cells but increased in Panc03.27 cells. V-ATPase inhibition again had no effect (Figure 3D). Employing the amino acid analogue N-methylaminoisobutyric acid (MeAIB), and L-threonine, which are known substrates of SLC38A1 and SLC1A5, respectively, we identified SLC1A5 as main import transporter in our cells. While competition of L-[^3^H]-glutamine with MeAIB did not affect the uptake of glutamine, competition with L-threonine almost completely blocked uptake (Figure 3D). Taken together, we identified SLC1A5 as the main amino acid importer in our cells, which is upregulated upon starvation, supposedly to increase nutrient uptake and maintain intracellular energy generation constant.

There is evidence in the literature, that apart from glutamine uptake via SLC transporters, glutamine supply can also be sustained break-down or proteins to amino acids within the lysosome. These amino acids can be exported into the cytosol by SLC38A7 (17) and subsequently utilized to maintain energy generation. Analyzing intracellular fluorescence of a transferrin-AlexaFluor™488 conjugate as a model substrate, reveals an increased internalization upon glutamine starvation (Figure 3E). Additionally, we found an increase in SLC38A7 mRNA (Figure 3A), which might hint to a SLC38A7-mediated compensation mechanism upon glutamine deprivation. However, siRNA-mediated knock-down of SLC38A7 did not increase the effect of concomitant glutamine starvation and V-ATPase inhibition (Figure 3F, Supplementary Figure 3, and Supplementary Table 2). Further analysis of glutamine metabolism, i.e., expression levels of glutaminolysis-associated enzymes, revealed no differences (Figure 3G) that could account for the missing therapeutic benefit of combining glutamine deprivation with V-ATPase inhibition.

**FIGURE 4:**
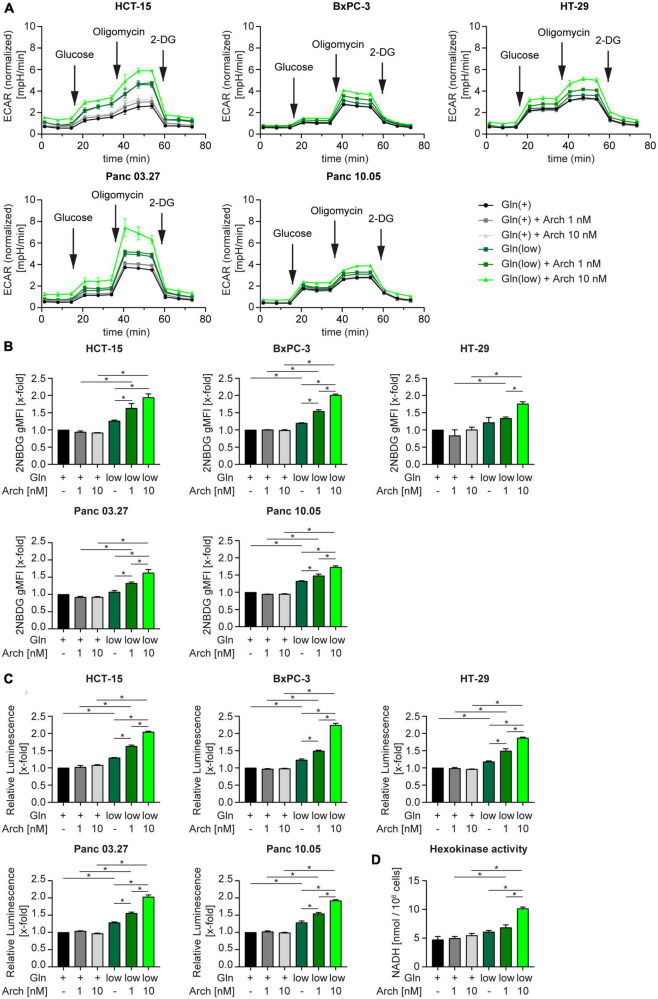
Metabolic shift toward glycolysis. **(A)** Cells were subjected to extracellular flux analysis on a Seahorse XFe96 device. Cells were pre-incubated as indicated for 24 h, prior to glycolysis stress test. Metabolic profiling was performed after 1 h of glucose starvation to assess basal glycolytic activity. This was followed by sequential injection of D-glucose and oligomycin to determine glycolysis and glycolytic capacity after injection of saturating concentrations of glucose and glycolytic reserve upon inhibition of oxidative phosphorylation, respectively. The last injection of the glycolysis inhibitor 2-deoxy-D-glucose (2-DG) served as control. ECAR is displayed over time as mean ± SEM. ECAR was normalized to cell number. **(B)** Cells were treated as indicated for 24 h followed by 2-NBDG staining. 2-NBDG intensity was assessed by flow cytometry analysis and displayed as geometric mean of fluorescent intensity (gMFI). **(C)** Lactate production was measured using Lactate-Glo™ assay kit. Luminescence correlates with amount of lactate. **(D)** HCT-15 cells were treated as indicated for 24 h followed by determination of Hexokinase activity. **(B–D)** Bar graphs display mean ± SEM (n = 3), Two-way ANOVA followed by Tukey’s multiple comparison test. *p < 0.05. ECAR (extracellular acidification rate).

We then posed the hypothesis, that cells undergo a metabolic shift from glutaminolysis to glycolysis when challenged with V-ATPase inhibition under glutamine-deprived conditions. A Seahorse™ glycolytic stress test revealed an increase in the extracellular acidification rate (ECAR) in all cell lines, when challenged with glutamine deprivation alone and together with archazolid treatment (Figure 4A). Subsequent addition of glucose, to induce glycolysis, oligomycin, to block mitochondrial respiration and therefore forcing the cells to fully rely on glycolysis, and 2-DG, to completely shut down glycolysis, allows assessment of glycolytic parameters. Upon glutamine deprivation in combination with V-ATPase inhibition, cells have an increased glycolytic capacity and glycolytic reserve (Supplementary Figure 4). By using CYRIS^®^ flox, a novel real-time platform to analyze cellular metabolism, we could verify our observation that cells have an increased glycolytic capacity and glycolytic reserve upon glutamine deprivation, which were both even enhanced upon V-ATPase inhibition (Supplementary Figures 5A, B). To confirm the metabolic shift toward glycolysis, we assessed glucose uptake and lactate production in our cells. Analyzing the uptake of fluorescent glucose analogue 2-NBDG by flow cytometry revealed an increased substrate uptake upon glutamine deprivation (Figure 4B). Along the line, lactate production increased likewise (Figure 4C). We additionally analyzed several glycolytic key enzymes but found no significant change in expression (Supplementary Figure 5C and Supplementary Table 3). As enzyme activity does not necessarily correlate with expression, we assessed the combined activity of all hexokinase isoforms upon glutamine deprivation in combination with archazolid treatment. Hexokinases catalyze the phosphorylation of glucose to glucose-6-phosphate, which represents the first, obligatory, and rate-limiting step of glycolysis. Upon glutamine deprivation or V-ATPase inhibition, Hexokinase activity did not significantly change, however, upon a combination of both, enzyme activity greatly increases (Figure 4D). This further strengthens the finding that glycolysis is induced to maintain cellular metabolism when challenged with glutamine deprivation and inhibition of autophagy.

## 4. Discussion

Metabolic reprogramming, like increased reliance on glutamine metabolism, is an established hallmark of cancer cells, which is an intensively investigated anti-cancer treatment strategy (22, 23). In this study, we aimed to enhance the therapeutic effectiveness of glutamine deprivation by co-inhibiting the V-ATPase. Glutamine starvation induced pro-survival autophagy, which could be blocked by V-ATPase inhibition. However, we could not observe a synergistic anti-cancer effect. Mechanistically, cells upregulate glycolysis which we conclude contributes to the maintenance of survival.

Cellular nutrient sensing is centered at lysosomes, which therefore regulate metabolic adaptations in accordance with nutrient availability, by catabolic autophagy pathways (11, 24). However, the exact role of autophagy upon starvation is still debated and differs with the diminished nutrients. Concerning glutamine deprivation, there is accumulating evidence, that lysosome-dependent pro-survival autophagy is induced. For instance, Yu et al. (25) show that deprivation of glutamine and serum leads to a rapid fusion of lysosomes with autophagosomes to form large autolysosomes in a variety of cell lines across different species, indicating that degradation of lysosomes is an evolutionarily conserved response to starvation. In line with that, Mukha et al. (26) report that ATG5-dependent autophagy is a pro-survival response upon glutamine deprivation of prostate cancer cells. Additionally, others have shown that also glucose starvation induces ATG5 and LC3-dependent microautophagy in cancer (27). With our study, we can add evidence that glutamine starvation leads to significant acidification of lysosomes accompanied by autophagy induction, as evident by a decrease in LC3-II level, which goes in hand with a decreased mTORC1 activity (Figure 1F). In contrast to these findings, an elevation of lysosomal pH following serum starvation was reported, which did not impact autophagy (28). In addition, Wilden et al. (29) found that starvation with Hank’s Balanced Salt Solution (HBSS) does not affect lysosomes or autophagy induction. Yet, when they simultaneously disrupted lysosomal function with chloroquine, they observed large vacuoles, which could be dissipated by the re-addition of glutamine. Interestingly, Chiodi et al. (30) further report autophagy induction by glucose starvation, but not serum or glutamine starvation, in human fibroblasts. Taken together, our results support evidence in the literature, that glutamine starvation induces LC3-dependent autophagy in cancer cells. However, whether autophagy induction is a conserved response to any lack of nutrients is still a matter that requires further investigation.

It is therefore a reasonable therapeutic approach to block these adaptations simultaneously with glutamine deprivation. There are to date many pre-clinical and clinical studies available, that show a beneficial effect of combining glutaminase inhibitors with additional therapeutic strategies. In this regard, several studies in a broad variety of tumor entities have shown synergistic effects of glutaminase inhibitors with frequently used cytostatics (31–35). Other studies have even found that glutaminase inhibition can overcome therapy resistance for instance in prostate cancer cells, which can be re-sensitized to radiation therapy (26), or methotrexate. Another study reports that glutamine deprivation in the core regions of tumors contributes to the formation of cancer stemness, which can be inhibited by co-inhibition of mitochondrial fission (36). As our results demonstrate that glutamine deprivation induces autophagy (Figure 1), we investigated the therapeutical benefit of a combination of glutaminase inhibitors with the V-ATPase inhibitor archazolid, a known anti-cancer compound that inhibits autophagy. However, we found no beneficial effect.

In contrast to our observation, several reports in the literature describe a synergistic effect of glutaminase inhibition and inhibition of lysosomal function. This was shown in colorectal cancer cells for a combination of glutaminase inhibitor 968 with the lysosomotropic compound chloroquine (36–38). Similar results were described by Seo et al. (39) who could show that in pancreatic adenocarcinoma, glutamine, but not glucose, deprivation in combination with chloroquine resulted in a tremendous induction of cell death. In our study, however, glutamine deprivation in combination with autophagy inhibition did not lead to an augmentation in proliferation inhibition or apoptosis except for the effect of a BPTES and archazolid combination in HCT-15 cells (Figure 2). As this result does not fall in line with starvation or CB-839 treatment and data of the other cell lines, we figured an alternative mode of action could be underlying. Archazolid is a known substrate of multidrug resistance transporter P-glycoprotein (P-gp) (40), which is highly expressed in HCT-15 cells (41). Since we found that BPTES blocks P-gp activity, we conclude that the additive effect we observed by combining archazolid and BPTES rather results from increased cellular drug availability of archazolid than from glutaminase inhibition (Supplementary Figure 2).

Curious as to why we only detected moderate effects of glutamine deprivation and simultaneous autophagy inhibition in our model, we hypothesized, that when challenged with these conditions, cells can further adapt their metabolism to use other nutrients to ensure survival. There is evidence, that for instance exogenous asparagine or alanine might be used as alternative substrates in glutamine deprived conditions (37, 42, 43). Extracellular alanine, like glutamine, is taken up by SLC1A5 in exchange for export of arginine (44), hence SLC1A5 could play an important role in switching metabolism. Our results identify SLC1A5 as the main amino acid transporter in our model, which is activated upon starvation, however, it is also trapped in intracellular vesicles upon glutamine deprivation in combination with archazolid (Figure 3). This indicates, that increased uptake of exogenous amino acids might not be the primary metabolic escape of our cells. Interestingly, Liu et al. (45) discovered a close interaction between glutaminolysis and glycolysis in glioblastoma. In essence, they discovered a MTOR2/C-MYC/GFAT1 axis, which is responsible for a cross-talk between these pathways and is switched on and off to adapt to substrate availability. It is noteworthy, that the V-ATPase has been controversially implicated in glucose metabolism. While it was shown that the V-ATPase inhibitor archazolid leads to an upregulation of glucose uptake (46), others report that knock-down of the V-ATPase reduced glucose uptake and decreased extracellular acidification rate, indicating glycolysis inhibition (47). Our results on the other hand show, that V-ATPase inhibition alone does not yet influence extracellular acidification rate or glycolytic parameters in a glycolysis stress test significantly, but moderately induce glucose uptake and lactate production. Yet, when cells that are subjected to glutamine deprivation in combination with archazolid, they undergo a metabolic shift toward glycolysis, as evident by an increased glycolytic capacity and reserve, as well as strongly increased glutamine uptake an lactate production (Figure 4). Supporting this finding, we also detected an increase in activating phosphorylation of AMPK activity (Figure 1E) upon starvation, which is known to promote glycolysis (48). Along the line with our previous reports, archazolid did not further affect AMPK phosphorylation (10). We conclude that this metabolic adaptation to increased glucose reliance can at least partly restore cellular survival capacity, thereby preventing a beneficial effect of combining glutamine deprivation with archazolid. Furthermore, our report indicates an considerable metabolic plasticity, as we previously reported an increased glutamine dependency of cancer cells upon V-ATPase inhibition (49), which can, as we describe in this study, be reversed to increased reliance on glycolysis. Whether further metabolic routes contribute to metabolic plasticity in response to different treatment strategies is an interesting subject for future research.

## Data availability statement

The original contributions presented in this study are included in the article/Supplementary material, further inquiries can be directed to the corresponding author.

## Author contributions

FL and AG performed the experiments and analyzed data. FG and KB wrote the manuscript. FL, FG, AV, KS, and KB contributed to the conception and design of the study. KB supervised the project. All authors contributed to the manuscript revision, read, and approved the submitted version.
