# Supplementary material for: A metabolic shift toward glycolysis enables cancer cells to maintain survival upon concomitant glutamine deprivation and V-ATPase inhibition
Source: Front Nutr. 2023 May 15;10:1124678. doi: 10.3389/fnut.2023.1124678 (PMC10225586; doi:10.3389/fnut.2023.1124678)
Supplement: Supplementary file 1 [file Data_Sheet_1.pdf]

Supplementary Material

Induction of glycolysis circumvents V-ATPase dependent glutamine dependency

Florian Lengauer, Franz Geisslinger, Antje Gabriel, Karin von Schwarzenberg, Angelika Vollmar, Karin Bartel*

Ludwig-Maximilians University, Department Pharmacy, Pharmaceutical Biology, Munich, Germany

*** Correspondence:**
Dr. Karin Bartel
Karin.bartel@cup.uni-muenchen.de

1 Supplementary Methods

1.1 Cell culture

MCF-7 mammary gland adenocarcinoma cells, T24 urinary bladder cancer cells and HeLa cervical carcinoma cells were obtained from German Research Centre of Biological Material (DSMZ, Braunschweig, Germany). The liver cancer cell line HUH7 was obtained from ATCC. Short tandem repeat analysis and testing for mycoplasma contamination were performed regularly. Cells were grown in DMEM supplemented with 10% fetal calf serum (FCS) and cultured under constant humidity at 37 °C, 5% CO_2. Verapamil and elacridar were obtained from Selleck Chemical GmbH (Houston, USA).

1.2 Calcein Retention Assay

Calcein-AM is a P-glycoprotein (P-gp) substrate and can be actively transported from the plasma membrane by P-gp. Once it has entered the cytosol, it is cleaved by cellular esterases and becomes fluorescent. Thus, calcein-AM can be used to monitor P-gp activity. The protocol was adapted from Robey et al [1]. 120×10^3 cells/well (HCT-15) or 170×10^3 cells/well (BxPC-3) were seeded into 24-well plates, allowed to adhere overnight Subsequently, calcein-AM (Biomol GmbH, Hamburg, Germany) (200 nM) and the potential P-gp inhibitors (elacridar, verapamil served as positive controls) were added and incubated for 30 min at 37 °C protected from light. After incubation, cells were centrifuged (400 g, 5 min), washed with PBS and resuspended in 0.5 mL DMEM without phenol red (PAN Biotech GmbH, Aidenbach, Germany) containing the respective concentration of the P-gp inhibitor. Following a 60 min incubation time at 37 °C, calcein fluorescence was analyzed by flow cytometry using a FACSCanto™ II (Becton Dickinson GmbH, Heidelberg, Germany) and FACSDiva™ software (Becton Dickinson GmbH, Heidelberg, Germany). For data evaluation the flow cytometry analysis software FlowJo 10.8.1 (Becton Dickinson GmbH, Heidelberg, Germany) was employed.

1.3 Metabolic stress test

After treatment for 24 h on a sensor plate, media reservoirs were filled with basic measuring media (DMEM, supplemented with 4.5 g/L glucose, pH 7.2) supplemented with 584 mg/L (Gln(+)) or 116.8

mg/L (Gln(low)) glutamine and 10 % FCS. Medium was replaced with respective measuring media, the plate was sealed and loaded into the CYRIS® flox (INCYTON® GmbH, Planegg, Germany) (22). 1 µM Oligomycin, and 0.5 mM 2-Deoxyglucose (2-DG) were applied sequentially (4 cycles of 12 min each). The obtained raw data was normalized to cell density (crystal violet staining).

2 Supplementary Figures and Tables

2.1 Supplementary Figures

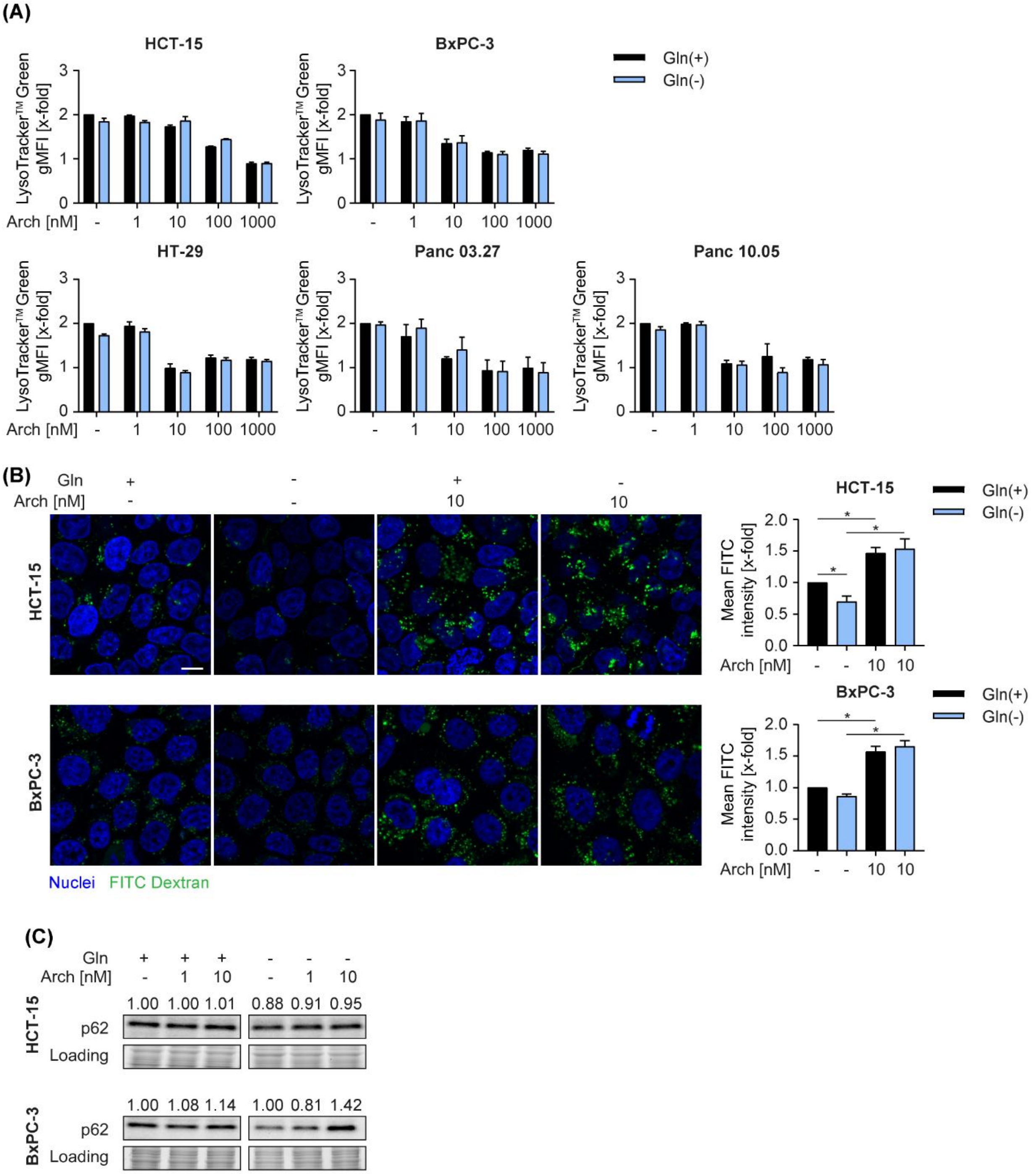

Supplementary Figure 1. (**A**) Cells were treated as indicated for 24 h followed by LysoTracker^{TM} Green FM staining. LysoTracker^{TM} Green FM intensity was assessed by flow cytometry analysis and displayed as geometric mean of fluorescent intensity (gMFI). (**B**) Cells were loaded with 200 µg/mL of FITC-Dextran 20 kDa (green) for 24 h. Subsequently cells were treated as indicated for 1 h before staining with Hoechst 33342 (nuclei, blue). FITC Dextran intensity was assessed by confocal microscopy. Scale bar: 10 µM. One representative image is shown. Quantification of mean FITC intensity of five independent images per condition was performed by ImageJ software. (**C**) p62 protein level in HCT-15 and BxPC-3 cells analyzed by western blotting after 24 h of treatment as indicated accompanied by quantification. One representative Western Blot is shown. Mean values are depicted above the respective bands. (**A,B**) Bar graphs display mean ± SEM (n=3), Two-way ANOVA followed by Tukey´s multiple comparison test, *p<0.05.

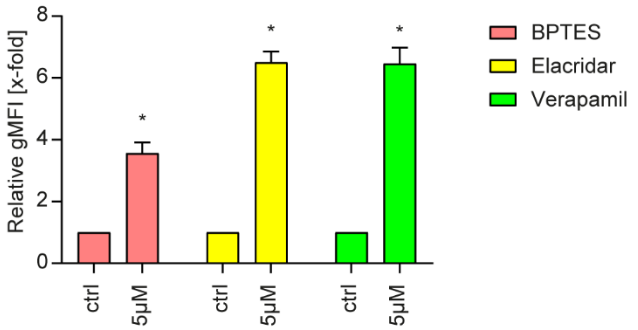

Supplementary Figure 2. Retention of the P-gp model substrate calcein-AM in HCT-15 cells was determined by flow cytometry. Cells were incubated with calcein-AM, in presence or absence of BPTES and the positive controls verapamil and elacridar at the indicated concentrations. Bar graph displays mean ± SEM (n=3), unpaired t-test with Welch´s correction, *p<0.05.

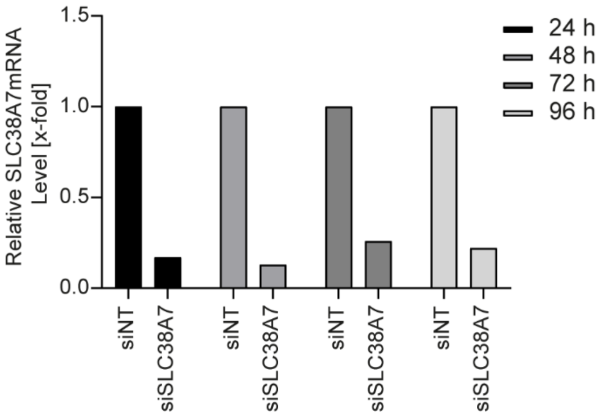

Supplementary Figure 3. Transfection control of SLC38A7 knockdown. The expression of glutamine transporters was analyzed by qPCR after the indicated times of knockdown.

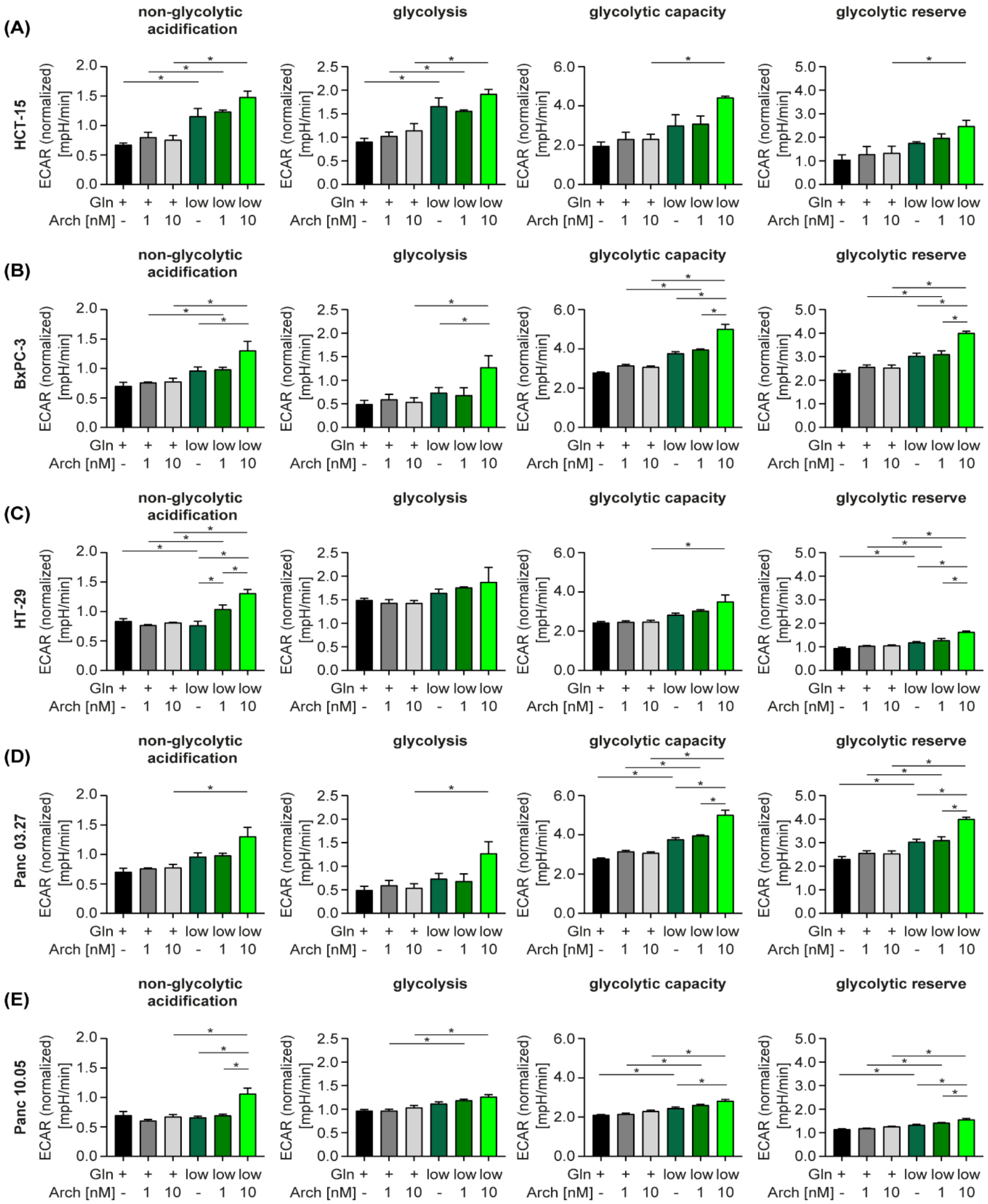

Supplementary Figure 4. (**A-E**) Non-glycolytic acidification, glycolysis, glycolytic capacity and glycolytic reserve parameter calculated from glycolysis stress test of Figure 4A in HCT-15 (**A**), BxPC-3 (**B**), HT-29 (**C**), Panc 03.27 (**D**) and Panc 10.05 (**E**) cell line. Bar graphs display mean ± SEM (n=3), Two-way ANOVA followed by Tukey´s multiple comparison test, *$p<0.05$.

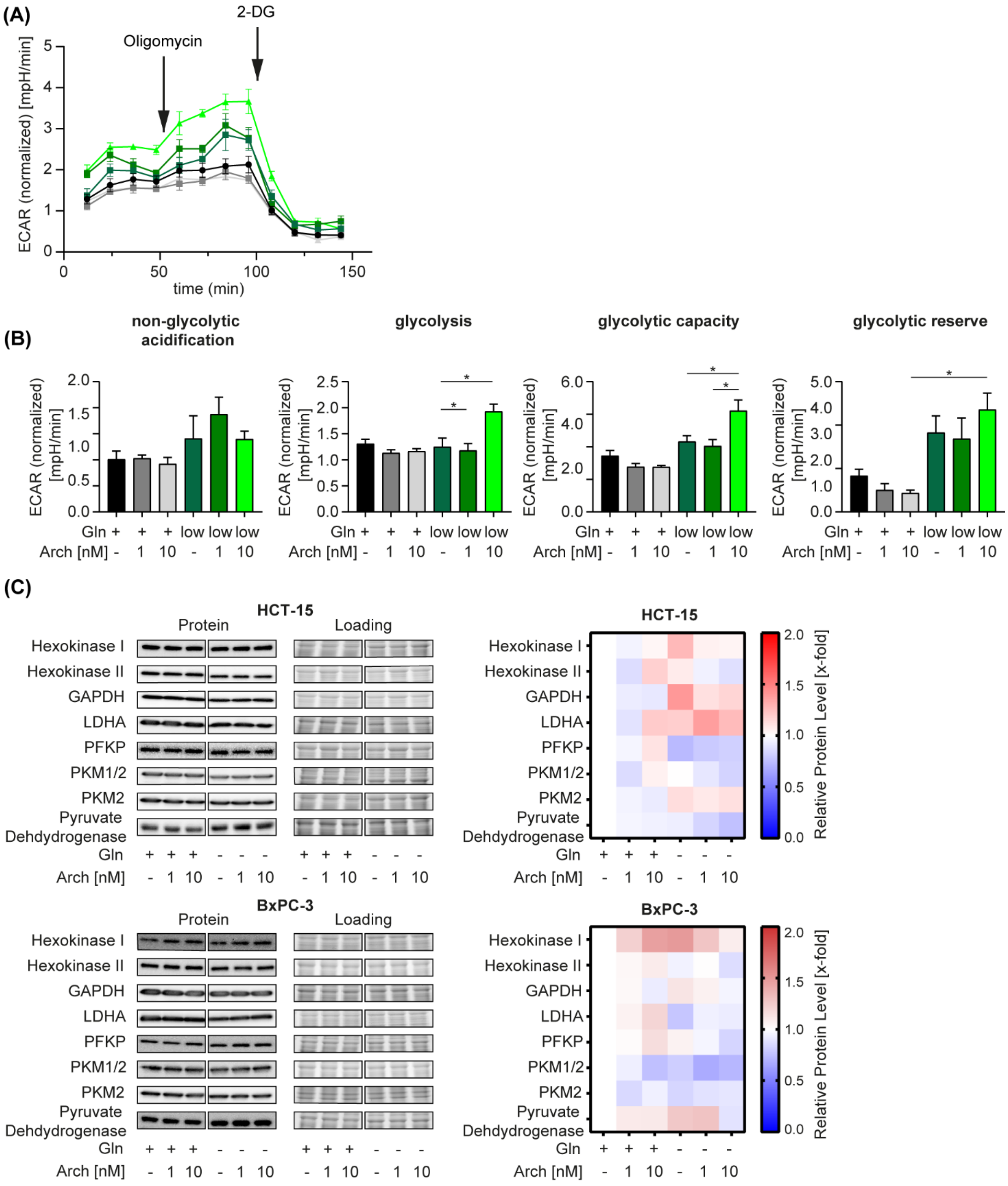

Supplementary Figure 5. (**A**) HCT-15 cells were subjected to extracellular flux analysis on a CYRIS® flox platform. Cells were pre-incubated as indicated for 24 h, prior to metabolic stress test. Metabolic profiling was performed directly after applying measuring medium containing D-glucose. This was followed by injection oligomycin to determine glycolytic capacity and glycolytic reserve upon inhibition of oxidative phosphorylation. The last injection of the glycolysis inhibitor 2-deoxy-D-glucose (2-DG) served as control. ECAR is displayed over time as mean ± SEM. ECAR was

normalized to cell number. (**B**) Non-glycolytic acidification, glycolysis, glycolytic capacity, and glycolytic reserve parameter calculated from glycolysis stress test of Figure S5A. Bar graphs display mean ± SEM (n=3), Two-way ANOVA followed by Tukey´s multiple comparison test, *p<0.05. (**C**) Glycolysis related enzyme protein level in HCT-15 and BxPC-3 cells analyzed by western blotting after 24 h of treatment as indicated. Heatmaps display median values (n=3). One representative Western Blot is shown.

3 Supplementary tables

3.1 Supplementary Table 1. Statistical evaluation of Figure 3A

**HCT-15**									
**Target**	**Gln(+) - vs. Gln(+) Arch 1 nM**	**Gln(+) - vs. Gln(+) Arch 10 nM**	**Gln(+) Arch 1 nM vs. Gln(+) Arch 10 nM**	**Gln(-) - vs. Gln(-) Arch 1 nM**	**Gln(-) - vs. Gln(-) Arch 10 nM**	**Gln(-) Arch 1 nM vs. Gln(-) Arch 10 nM**	**Gln(+) - vs. Gln(-) -**	**Gln(+) Arch 1 nM vs. Gln(-) Arch 1 nM**	**Gln(+) Arch 10 nM vs. Gln(-) Arch 10 nM**
**GLUL**	ns	ns	ns	ns	ns	ns	ns	*	*
**GLS1**	ns	ns	ns	ns	ns	*	ns	ns	*
**GLS2**	ns	ns	ns	ns	ns	ns	ns	ns	ns
**GOT1**	ns	ns	*	ns	ns	ns	*	*	*
**GOT2**	ns	ns	ns	ns	ns	ns	ns	ns	ns
**GDH1**	ns	ns	ns	ns	ns	ns	ns	ns	ns
**GDH2**	ns	ns	ns	ns	ns	ns	ns	ns	ns
**GPT2**	ns	ns	ns	ns	ns	ns	*	*	*
**BxPC-3**									
**Target**	**Gln(+) - vs. Gln(+) Arch 1 nM**	**Gln(+) - vs. Gln(+) Arch 10 nM**	**Gln(+) Arch 1 nM vs. Gln(+) Arch 10 nM**	**Gln(-) - vs. Gln(-) Arch 1 nM**	**Gln(-) - vs. Gln(-) Arch 10 nM**	**Gln(-) Arch 1 nM vs. Gln(-) Arch 10 nM**	**Gln(+) - vs. Gln(-) -**	**Gln(+) Arch 1 nM vs. Gln(-) Arch 1 nM**	**Gln(+) Arch 10 nM vs. Gln(-) Arch 10 nM**
**GLUL**	ns	ns	ns	ns	*	*	ns	ns	ns
**GLS1**	ns	ns	ns	ns	ns	ns	ns	ns	ns
**GLS2**	ns	ns	ns	ns	ns	ns	ns	ns	ns
**GOT1**	ns	ns	ns	ns	ns	*	ns	ns	ns
**GOT2**	ns	ns	ns	ns	ns	ns	ns	ns	ns
**GDH1**	ns	ns	ns	ns	*	*	ns	ns	ns
**GDH2**	ns	ns	ns	ns	ns	ns	ns	ns	ns
**GPT2**	ns	ns	ns	ns	ns	ns	*	*	*

Two-way ANOVA followed by Tukey´s multiple comparison test, *p<0.05, ns not significant

3.2 Supplementary Table 2. Statistical evaluation of Figure 3F

HCT-15									
**Target**	**Gln(+) - vs. Gln(+) Arch 1 nM**	**Gln(+) - vs. Gln(+) Arch 10 nM**	**Gln(+) Arch 1 nM vs. Gln(+) Arch 10 nM**	**Gln(-) - vs. Gln(-) Arch 1 nM**	**Gln(-) - vs. Gln(-) Arch 10 nM**	**Gln(-) Arch 1 nM vs. Gln(-) Arch 10 nM**	**Gln(+) - vs. Gln(-) -**	**Gln(+) Arch 1 nM vs. Gln(-) Arch 1 nM**	**Gln(+) Arch 10 nM vs. Gln(-) Arch 10 nM**
**GLUL**	ns	ns	ns	ns	ns	ns	ns	*	*
**GLS1**	ns	ns	ns	ns	ns	*	ns	ns	*
**GLS2**	ns	ns	ns	ns	ns	ns	ns	ns	ns
**GOT1**	ns	ns	*	ns	ns	ns	*	*	*
**GOT2**	ns	ns	ns	ns	ns	ns	ns	ns	ns
**GDH1**	ns	ns	ns	ns	ns	ns	ns	ns	ns
**GDH2**	ns	ns	ns	ns	ns	ns	ns	ns	ns
**GPT2**	ns	ns	ns	ns	ns	ns	*	*	*
**BxPC-3**									
**Target**	**Gln(+) - vs. Gln(+) Arch 1 nM**	**Gln(+) - vs. Gln(+) Arch 10 nM**	**Gln(+) Arch 1 nM vs. Gln(+) Arch 10 nM**	**Gln(-) - vs. Gln(-) Arch 1 nM**	**Gln(-) - vs. Gln(-) Arch 10 nM**	**Gln(-) Arch 1 nM vs. Gln(-) Arch 10 nM**	**Gln(+) - vs. Gln(-) -**	**Gln(+) Arch 1 nM vs. Gln(-) Arch 1 nM**	**Gln(+) Arch 10 nM vs. Gln(-) Arch 10 nM**
**GLUL**	ns	ns	ns	ns	*	*	ns	ns	ns
**GLS1**	ns	ns	ns	ns	ns	ns	ns	ns	ns
**GLS2**	ns	ns	ns	ns	ns	ns	ns	ns	ns
**GOT1**	ns	ns	ns	ns	ns	*	ns	ns	ns
**GOT2**	ns	ns	ns	ns	ns	ns	ns	ns	ns
**GDH1**	ns	ns	ns	ns	*	*	ns	ns	ns
**GDH2**	ns	ns	ns	ns	ns	ns	ns	ns	ns
**GPT2**	ns	ns	ns	ns	ns	ns	*	*	*

Two-way ANOVA followed by Tukey´s multiple comparison test, *p<0.05, ns not significant

3.3 Supplementary Table 3. Statistical evaluation of Figure S5C

HCT-15									
**Target**	**Gln(+) - vs. Gln(+) Arch 1 nM**	**Gln(+) - vs. Gln(+) Arch 10 nM**	**Gln(+) Arch 1 nM vs. Gln(+) Arch 10 nM**	**Gln(-) - vs. Gln(-) Arch 1 nM**	**Gln(-) - vs. Gln(-) Arch 10 nM**	**Gln(-) Arch 1 nM vs. Gln(-) Arch 10 nM**	**Gln(+) - vs. Gln(-) -**	**Gln(+) Arch 1 nM vs. Gln(-) Arch 1 nM**	**Gln(+) Arch 10 nM vs. Gln(-) Arch 10 nM**
**Hexo-kinase I**	ns	ns	ns	ns	ns	ns	ns	ns	ns
**Hexo-kinase II**	ns	ns	ns	ns	ns	ns	ns	ns	ns
**GAPDH**	ns	ns	ns	ns	ns	ns	ns	ns	ns
**LDHA**	ns	ns	ns	ns	ns	ns	ns	ns	ns
**PFKP**	ns	ns	ns	ns	ns	ns	ns	ns	ns
**PKM1/2**	ns	ns	ns	ns	ns	ns	ns	ns	ns
**PKM2**	ns	ns	ns	ns	ns	ns	ns	ns	ns
**Pyruvate Dehydro-genase**	ns	ns	ns	ns	ns	ns	ns	ns	ns

BxPC-3									
**Target**	**Gln(+) - vs. Gln(+) Arch 1 nM**	**Gln(+) - vs. Gln(+) Arch 10 nM**	**Gln(+) Arch 1 nM vs. Gln(+) Arch 10 nM**	**Gln(-) - vs. Gln(-) Arch 1 nM**	**Gln(-) - vs. Gln(-) Arch 10 nM**	**Gln(-) Arch 1 nM vs. Gln(-) Arch 10 nM**	**Gln(+) - vs. Gln(-) -**	**Gln(+) Arch 1 nM vs. Gln(-) Arch 1 nM**	**Gln(+) Arch 10 nM vs. Gln(-) Arch 10 nM**
**Hexo-kinase I**	ns	ns	ns	ns	ns	ns	ns	ns	ns
**Hexo-kinase II**	ns	ns	ns	ns	ns	ns	ns	ns	ns
**GAPDH**	ns	ns	ns	ns	ns	ns	ns	ns	ns
**LDHA**	ns	ns	ns	ns	ns	ns	ns	ns	ns
**PFKP**	ns	ns	ns	ns	ns	ns	ns	ns	ns
**PKM1/2**	ns	ns	ns	ns	ns	ns	ns	ns	ns
**PKM2**	ns	ns	ns	ns	ns	ns	ns	ns	ns
**Pyruvate Dehydro-genase**	ns	ns	ns	ns	ns	ns	ns	ns	ns

Two-way ANOVA followed by Tukey´s multiple comparison test, *$p<0.05$, ns not significant

3.4 Supplementary Table 4. Primary Antibodies

Antibody	Supplier	Product No	Dilution
**AMPKα**	Cell Signaling Technology Inc. (Danvers, USA)	2793	1:1000
**AMPKα phospho (Thr172)**	Cell Signaling Technology Inc. (Danvers, USA)	2535	1:1000
**GAPDH**	Cell Signaling Technology Inc. (Danvers, USA)	5174	1:1000
**Hexokinase I**	Cell Signaling Technology Inc. (Danvers, USA)	2024	1:1000
**Hexokinase II**	Cell Signaling Technology Inc. (Danvers, USA)	2867	1:1000
**LC-3 I & II**	Cell Signaling Technology Inc. (Danvers, USA)	4108	1:1000
**LDHA**	Cell Signaling Technology Inc. (Danvers, USA)	3582	1:1000
**SLC1A5**	Cell Signaling Technology Inc. (Danvers, USA)	8057	1:1000 (WB); 1:200 (Confocal Imaging)
**SLC38A1**	Merck Millipore kGaA (Darmstadt, Germany)	MABN502	1:1000 (WB); 1:200 (Confocal Imaging)
**P70S6K**	Cell Signaling Technology Inc. (Danvers, USA)	9202	1:1000
**P70S6K phospho (Thr389)**	Cell Signaling Technology Inc. (Danvers, USA)	9205	1:1000
**P62**	Cell Signaling Technology Inc. (Danvers, USA)	8025	1:1000
**PFKP**	Cell Signaling Technology Inc. (Danvers, USA)	8164	1:1000
**PKM1/2**	Cell Signaling Technology Inc. (Danvers, USA)	3190	1:1000
**PKM2**	Cell Signaling Technology Inc. (Danvers, USA)	4053	1:1000
**Pyruvate Dehydrogenase**	Cell Signaling Technology Inc. (Danvers, USA)	3205	1:1000

3.5 Supplementary Table 5. Secondary Antibodies

Antibody	Supplier	Product No	Dilution
**Goat-anti-mouse IgG (H+L) Alexa-Fluor™ 488**	Thermo Fisher Scientific Inc. (Waltham, USA)	A-11001	1:400
**Goat-anti-rabbit IgG (H+L) Alexa-Fluor™ 647**	Thermo Fisher Scientific Inc. (Waltham, USA)	A-21245	1:400
**HRP, Goat-Anti-Mouse IgG2b**	Southern Biotech Assoc. (Birmingham, USA)	1090-05	1:3000
**HRP, Goat-Anti-Rabbit IgG (H+L)**	Bio-Rad Laboraties Inc. (Hercules, USA)	172-1019	1:3000

3.6 Supplementary Table 6. Quantitative real-time PCR analysis primer sequences

Target	Primer	Sequence (5'-3')
**Actin**	FW	CCA ACC GCC AGA AGA TGA
**Actin**	RV	CCA GAG GCG TAC AGG GAT AG
**GDH1**	FW	AGG AAT GAC ACC AGG GTT TG
**GDH1**	RV	TCA GAC TCA CCA ACA GCA ATA C
**GDH2**	FW	CAC TCT GCC TTG GCA TAC AC
**GDH2**	RV	CTC AGG TCC AAT CCC AGG TT
**GLUL**	FW	CCT GCT TGT ATG CTG GAG TC
**GLUL**	RV	GAT CTC CCA TGC TGA TTC CT
**GLS1**	FW	GCT GTG CTC CAT TGA AGT GA
**GLS1**	RV	GCA AAC TGC CCT GAG AAG TC
**GLS2**	FW	ATC AGA AAG TGG CAT GCT GT
**GLS2**	RV	GCC TTT AGT GCA GTG GTG AA
**GOT1**	FW	CAA CTG GGA TTG ACC CAA CT
**GOT1**	RV	GGA ACA GAA ACC GGT GCT T
**GOT2**	FW	GTT TGC CTC TGC CAA TCA TAT G
**GOT2**	RV	GAG GGT TGG AAT ACA TGG GAC
**GPT2**	FW	GGA GCT AGT GAC GGC ATT TCT ACG A
**GPT2**	RV	CCC AGG GTT GAT TAT GCA GAG CA
**SLC38A1**	FW	GCA CCA CAG GGA AGT TCG TA
**SLC38A1**	RV	ACT ATC ACC ACC AGA ACG CG
**SLC38A2**	FW	GCA GTG GAA TCC TTG GGC TT
**SLC38A2**	RV	ATA AAG ACC CTC CTT CAT TGG CA
**SLC38A5**	FW	GAG AGG GTG CCC GAA CCT
**SLC38A5**	RV	CCT CGA AAT CCA TGA ACT GGA C
**SLC38A7**	FW	CCC CAG GGA GAT TGG TTT CC
**SLC38A7**	RV	GGT CTT CAC TTC AGG CTG CT
**SLC38A8**	FW	GAC CTC AGC GAG ATC GTC AG
**SLC38A8**	RV	AAG ATG AAG GTG CCG ACC AG
**SLC38A9**	FW	GCC ATC CTG ACA ACA GCT CT
**SLC38A9**	RV	GGA GGA GGA GCC CTA CAA GA
**SLC38A10**	FW	ATG ATG TCA GTG GCT GTG GG
**SLC38A10**	RV	CTG GGA GGA AAG TGC GTT CT
**SLC1A5**	FW	GAA CTC CCA GCT TTC GGA CA
**SLC1A5**	RV	CCT TGG AGT CTC GAG GAG GA

4 Supplementary References

1. Robey, R.W., et al., *Inhibition of P-glycoprotein (ABCB1)- and multidrug resistance-associated protein 1 (ABCC1)-mediated transport by the orally administered inhibitor, CBT-1((R)).* Biochem Pharmacol, 2008. **75**(6): p. 1302-12.
