# Supplementary material for: A metabolic shift toward glycolysis enables cancer cells to maintain survival upon concomitant glutamine deprivation and V-ATPase inhibition
Source: Front Nutr. 2023 May 15;10:1124678. doi: 10.3389/fnut.2023.1124678 (PMC10225586; doi:10.3389/fnut.2023.1124678)
Supplement: Supplementary file 2 [file Data_Sheet_2.pdf]

Supplementary Material

Induction of glycolysis circumvents V-ATPase dependent glutamine dependency

Florian Lengauer, Franz Geisslinger, Antje Gabriel, Karin von Schwarzenberg, Angelika Vollmar, Karin Bartel*

Ludwig-Maximilians University, Department Pharmacy, Pharmaceutical Biology, Munich, Germany

*** Correspondence:**
Dr. Karin Bartel
Karin.bartel@cup.uni-muenchen.de

1 Full Blots and original images

1.1 Figure 1C

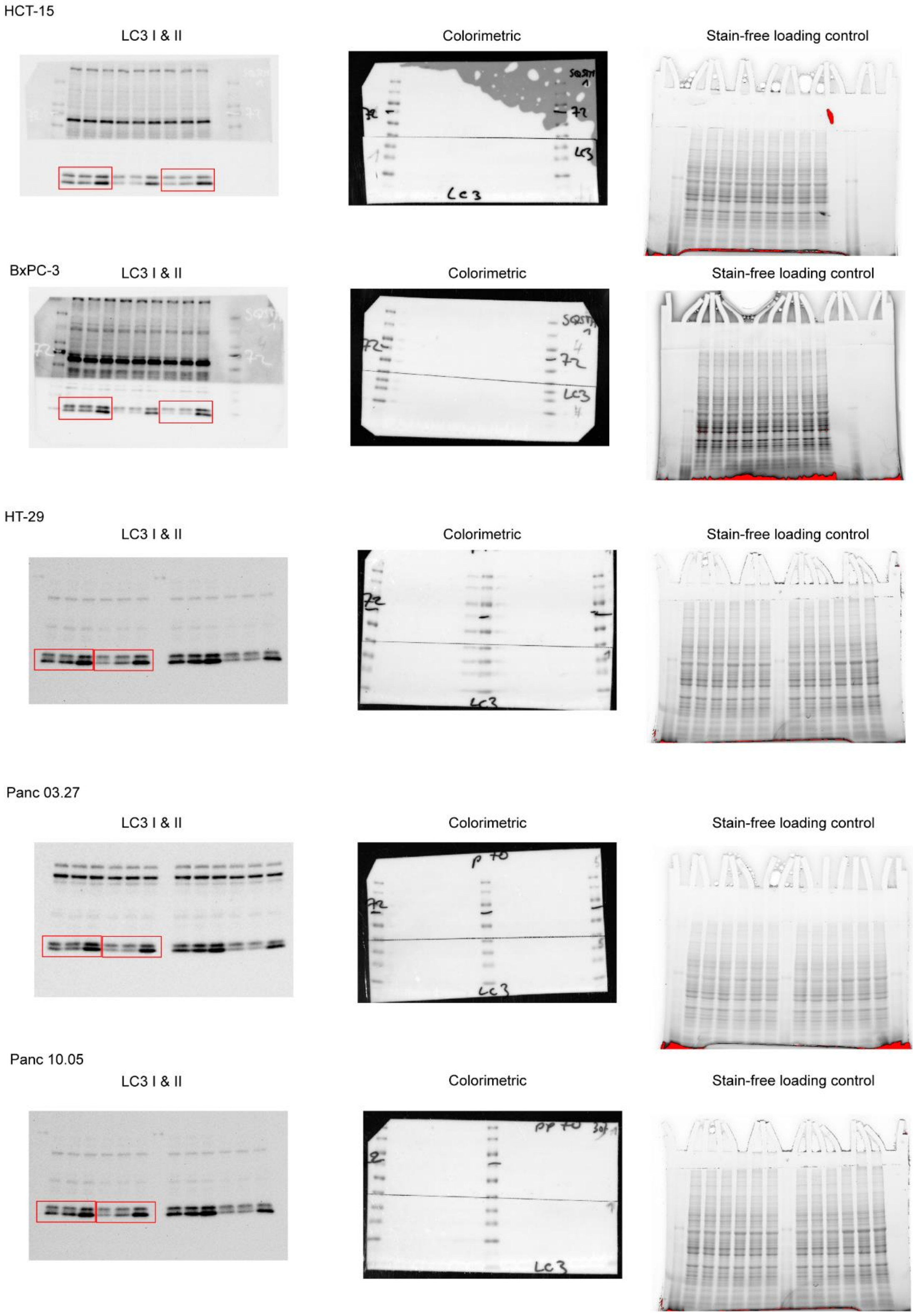

1.2 Figure 1D

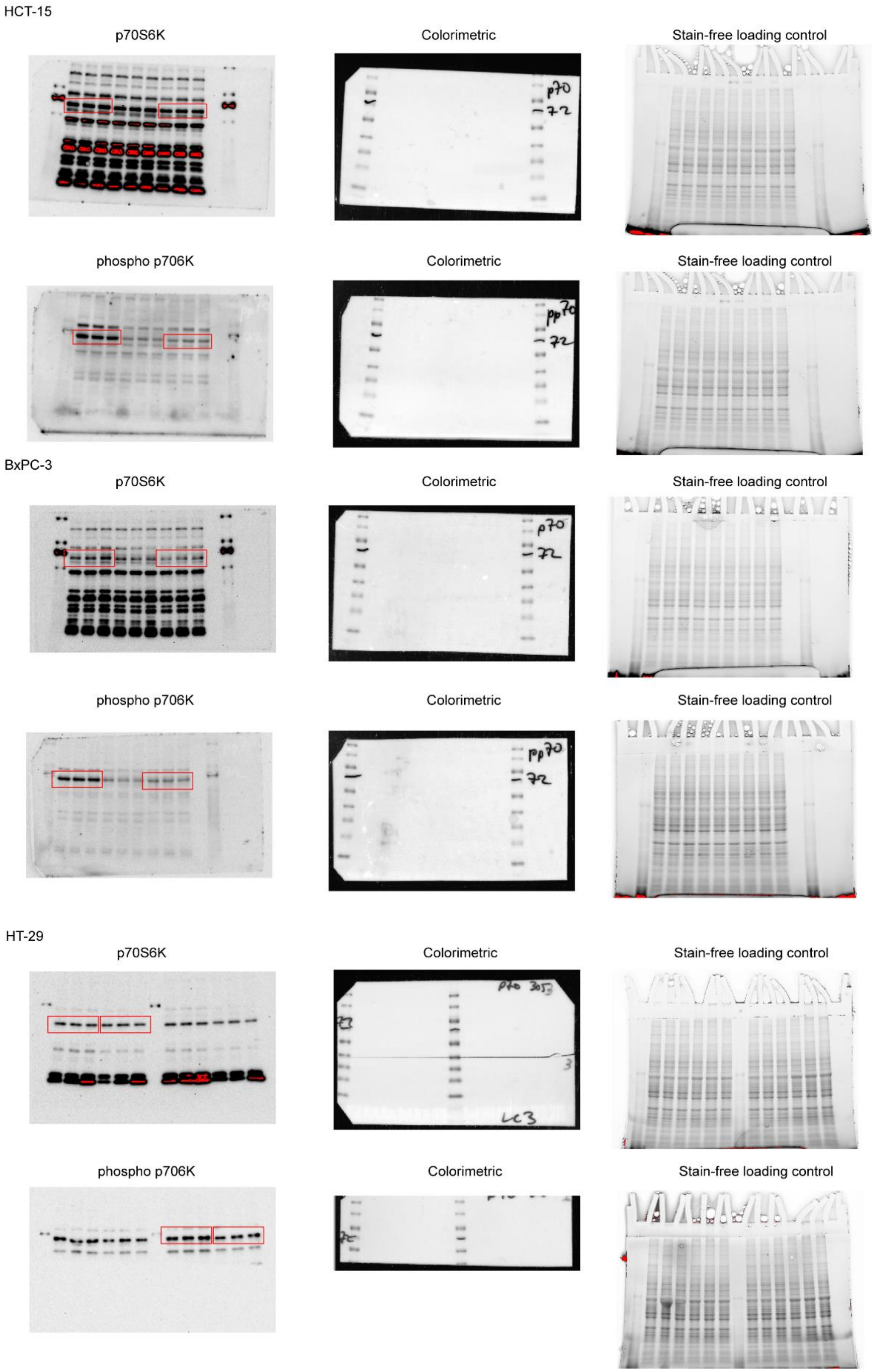

HCT-15
p70S6K
Colorimetric
Stain-free loading control
phospho p706K
Colorimetric
Stain-free loading control
BxPC-3
p70S6K
Colorimetric
Stain-free loading control
phospho p706K
Colorimetric
Stain-free loading control
HT-29
p70S6K
Colorimetric
Stain-free loading control
phospho p706K
Colorimetric
Stain-free loading control

Panc 03.27

p70S6K

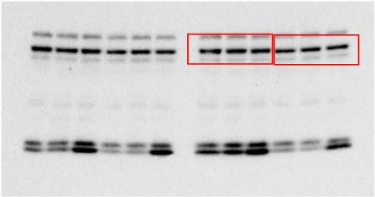

Colorimetric

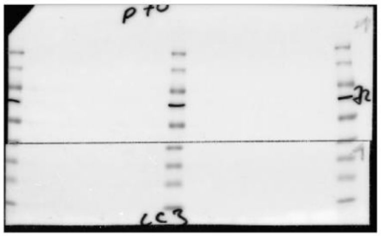

Stain-free loading control

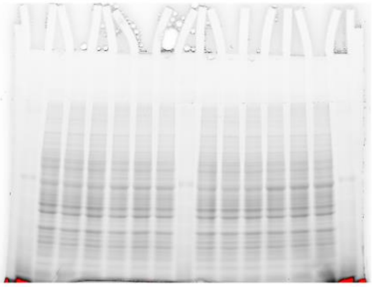

phospho p706K

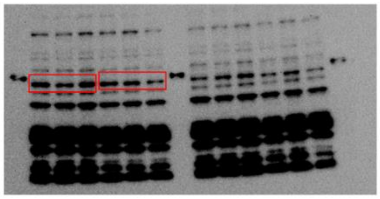

Colorimetric

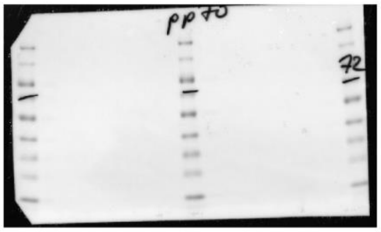

Stain-free loading control

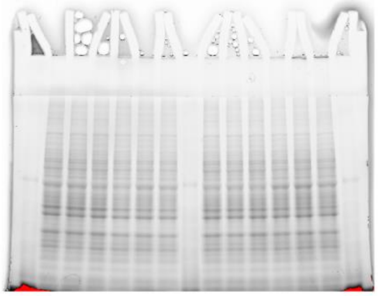

Panc 10.05

p70S6K

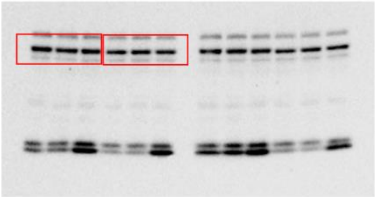

Colorimetric

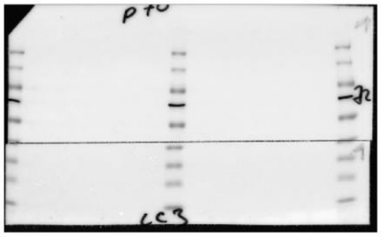

Stain-free loading control

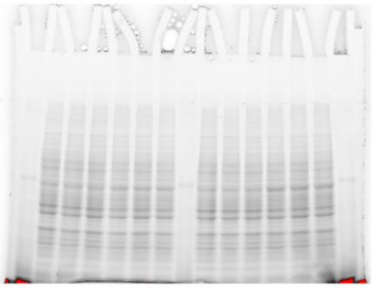

phospho p706K

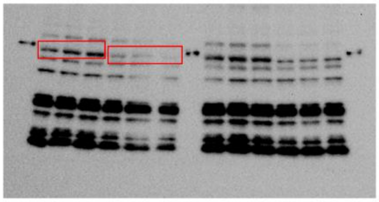

Colorimetric

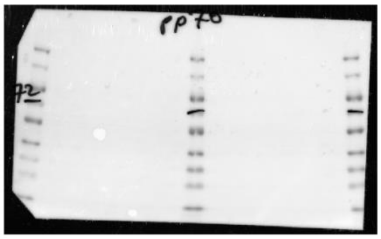

Stain-free loading control

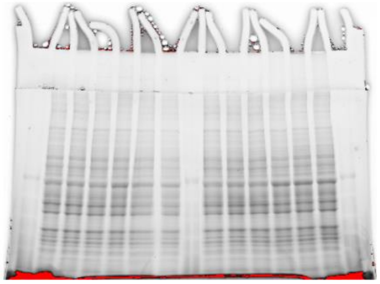

1.3 Figure 1E

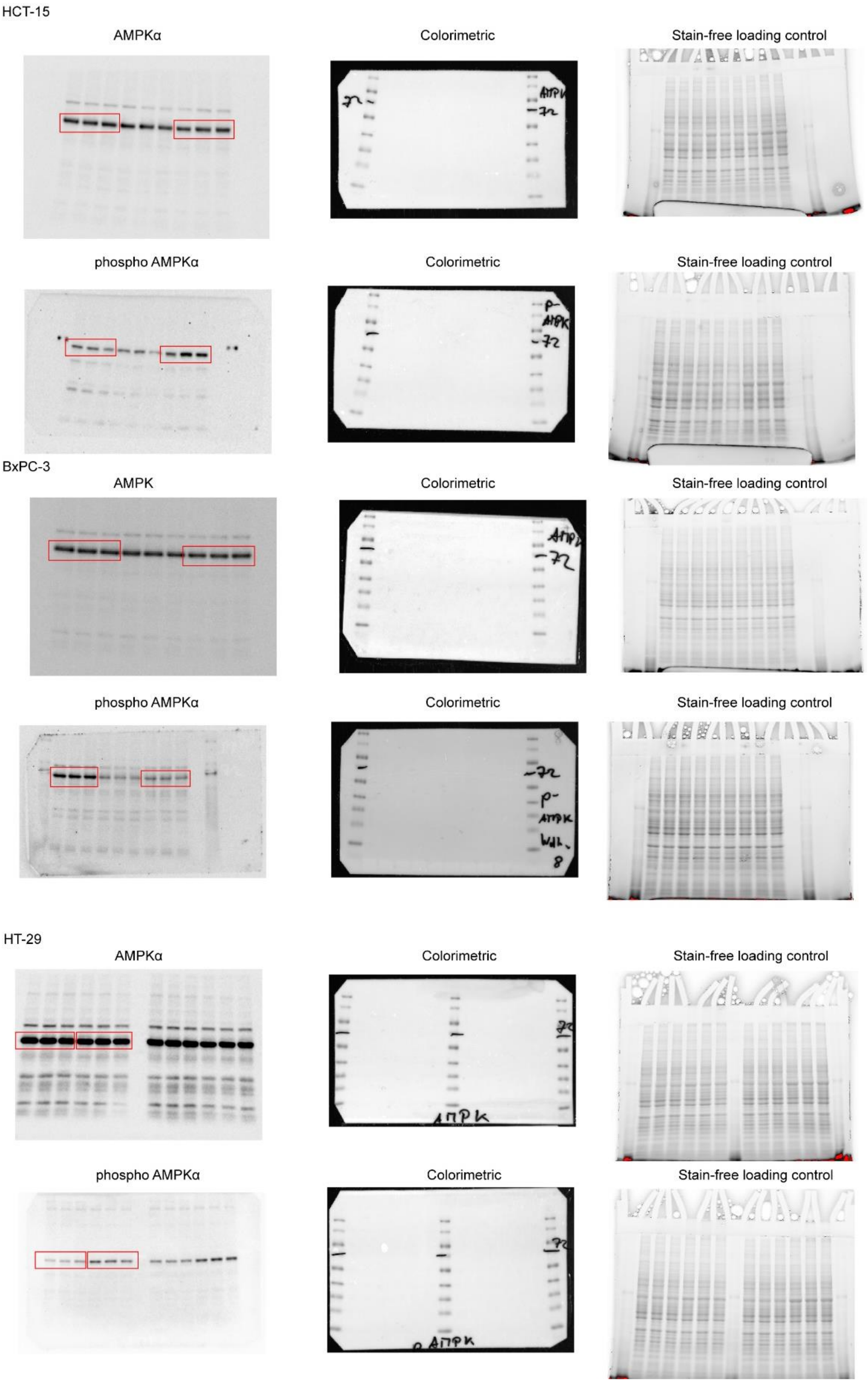
HCT-15
AMPKα
Colorimetric
Stain-free loading control
phospho AMPKα
Colorimetric
Stain-free loading control
BxPC-3
AMPK
Colorimetric
Stain-free loading control
phospho AMPKα
Colorimetric
Stain-free loading control
HT-29
AMPKα
Colorimetric
Stain-free loading control
phospho AMPKα
Colorimetric
Stain-free loading control

Panc 03.27

AMPKα

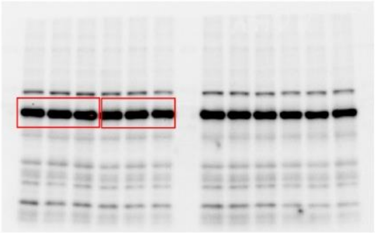

Colorimetric

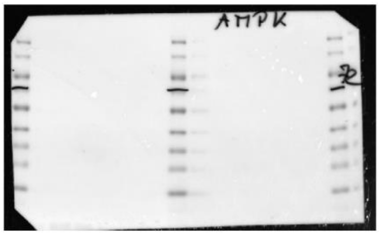

Stain-free loading control

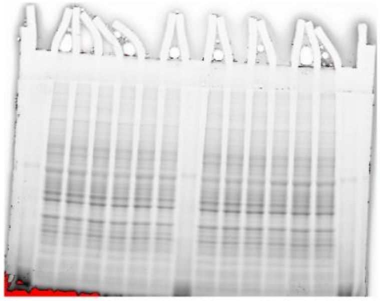

phospho AMPKα

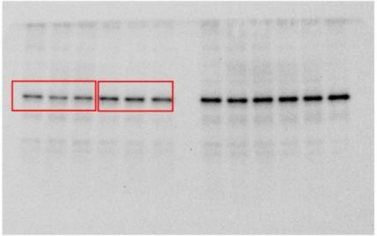

Colorimetric

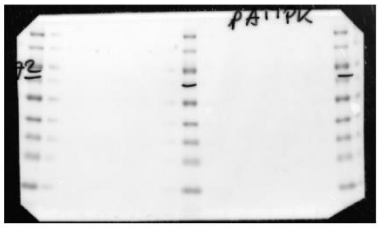

Stain-free loading control

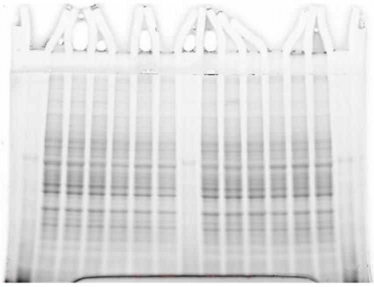

Panc 10.05

AMPK

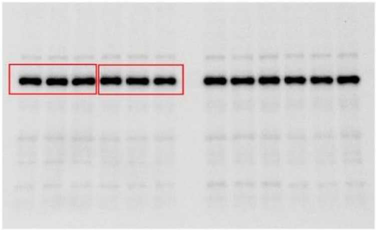

Colorimetric

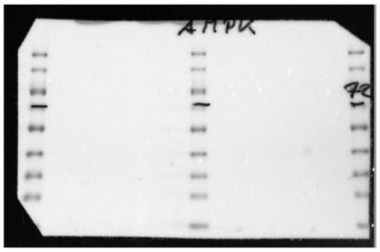

Stain-free loading control

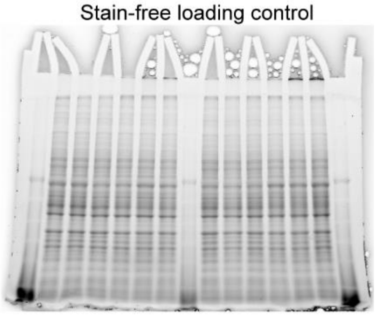

phospho AMPKα

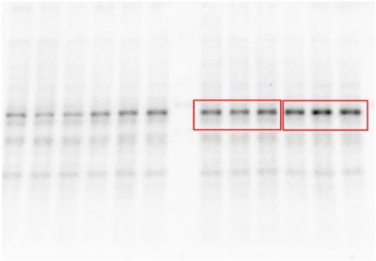

Colorimetric

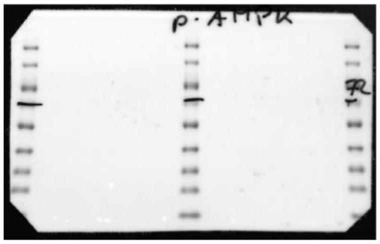

Stain-free loading control

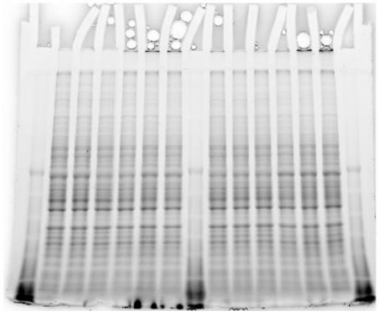

1.4 Figure 3B

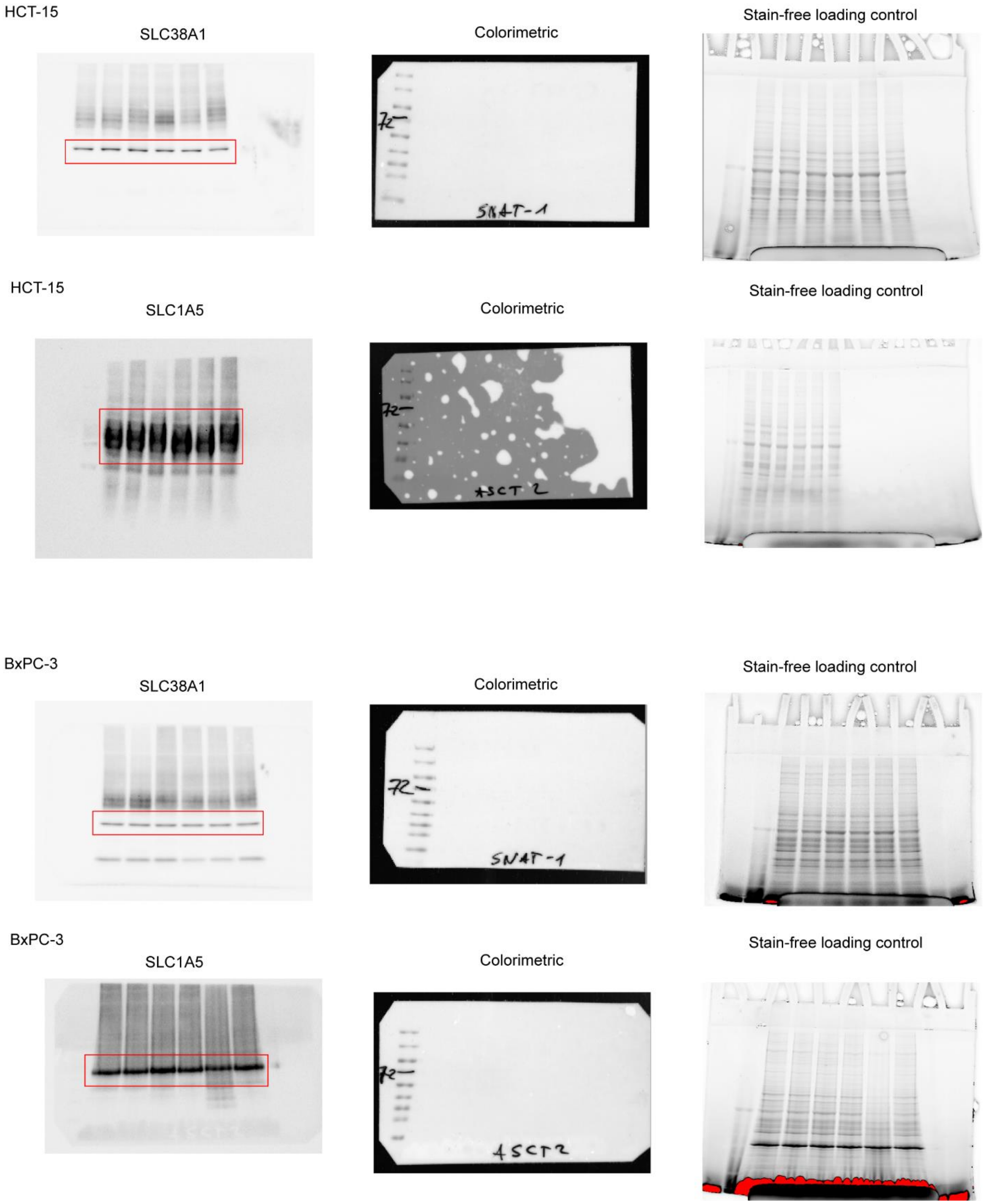
HCT-15
SLC38A1
Colorimetric
72
SNAT-1
Stain-free loading control
HCT-15
SLC1A5
Colorimetric
72
ASCT 2
Stain-free loading control
BxPC-3
SLC38A1
Colorimetric
72
SNAT-1
Stain-free loading control
BxPC-3
SLC1A5
Colorimetric
72
ASCT2
Stain-free loading control

1.5 Figure S1C

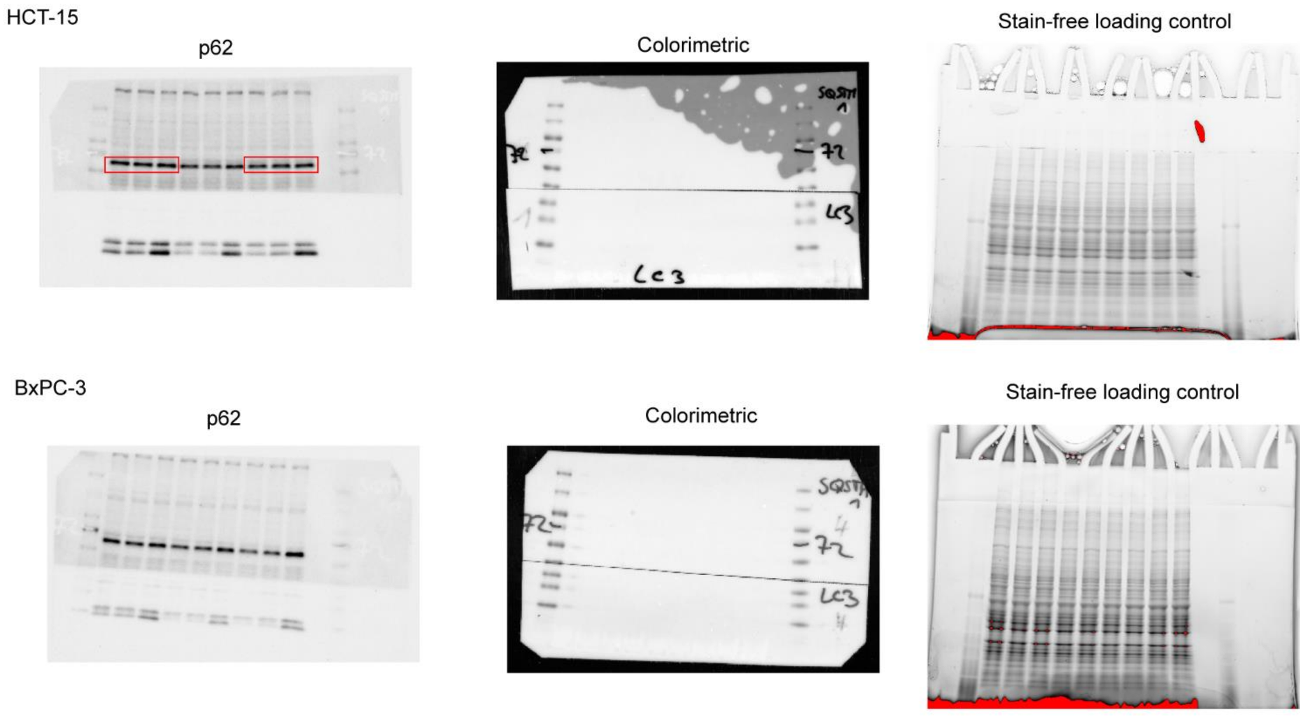

1.6 Figure S5C

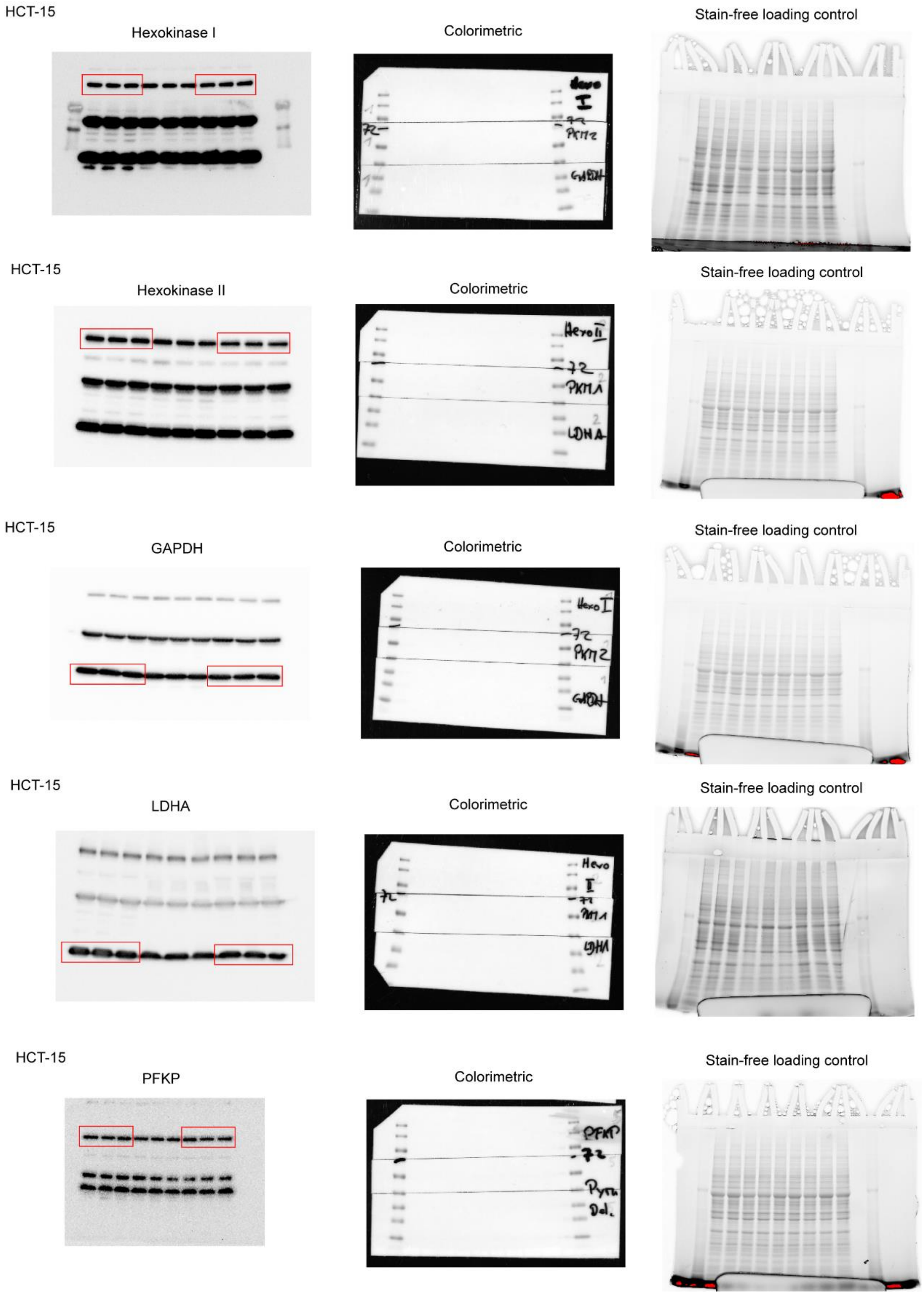

HCT-15

PKM1/2

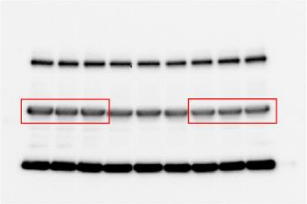

Colorimetric

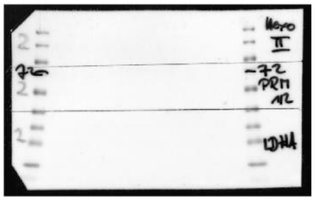

Stain-free loading control

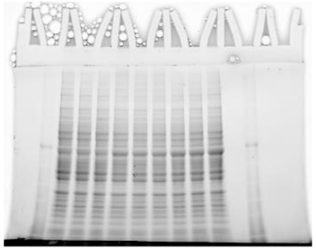

HCT-15

PKM2

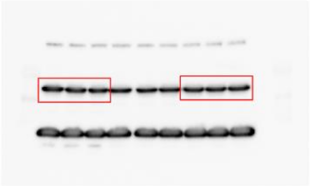

Colorimetric

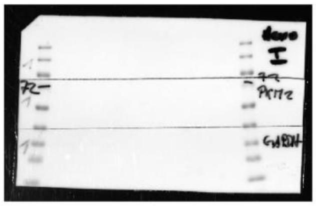

Stain-free loading control

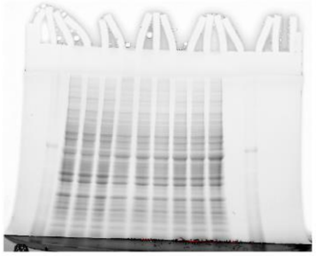

HCT-15

Pyruvate Dehydrogenase

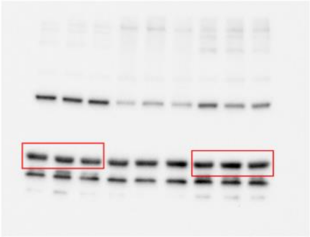

Colorimetric

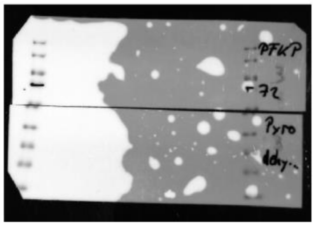

Stain-free loading control

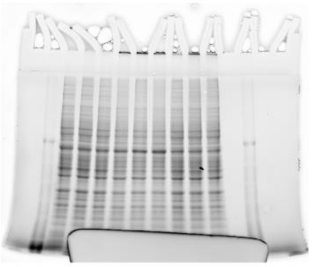

BxPC-3

Hexokinase I

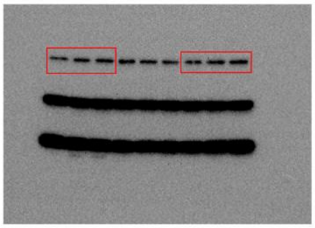

Colorimetric

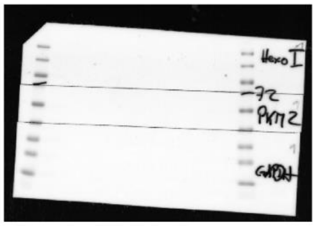

Stain-free loading control

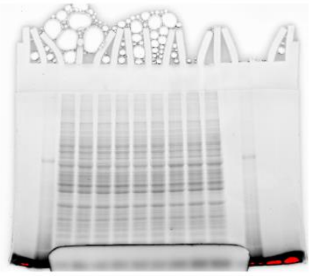

BxPC-3

Hexokinase II

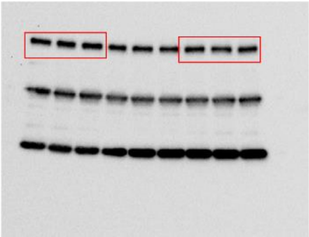

Colorimetric

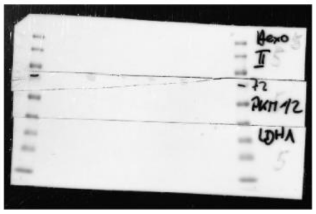

Stain-free loading control

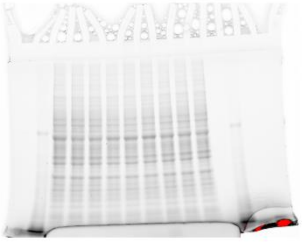

BxPC-3

GAPDH | Colorimetric | Stain-free loading control

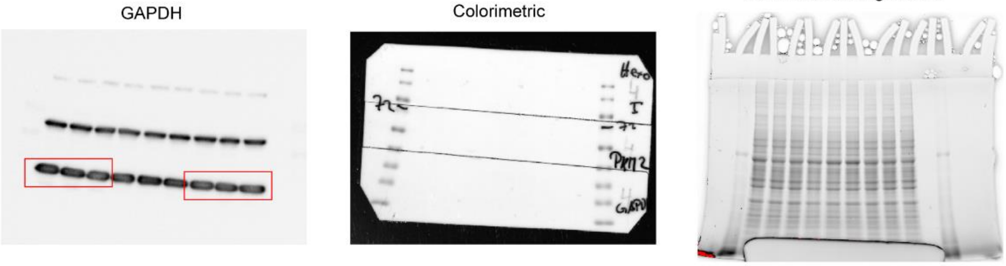

BxPC-3

LDHA | Colorimetric | Stain-free loading control

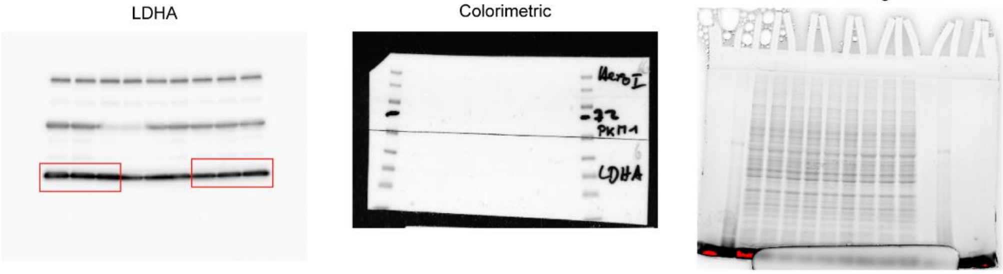

BxPC-3

PFKP | Colorimetric | Stain-free loading control

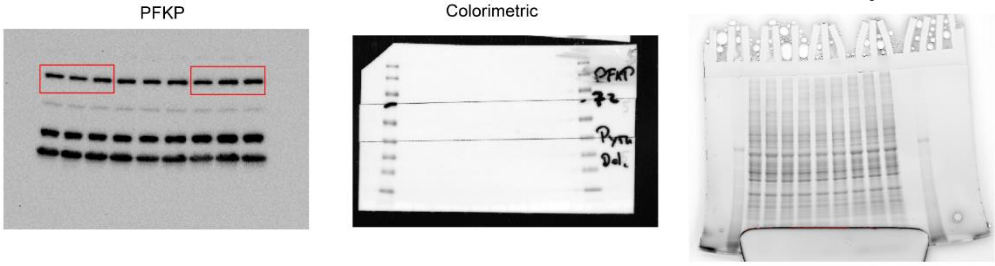

BxPC-3

PKM1/2 | Colorimetric | Stain-free loading control

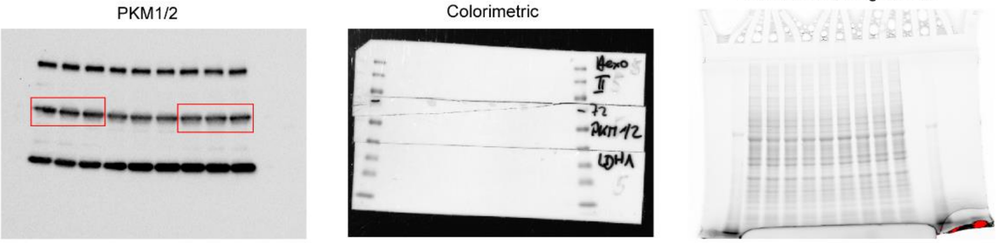

BxPC-3

PKM2 | Colorimetric | Stain-free loading control

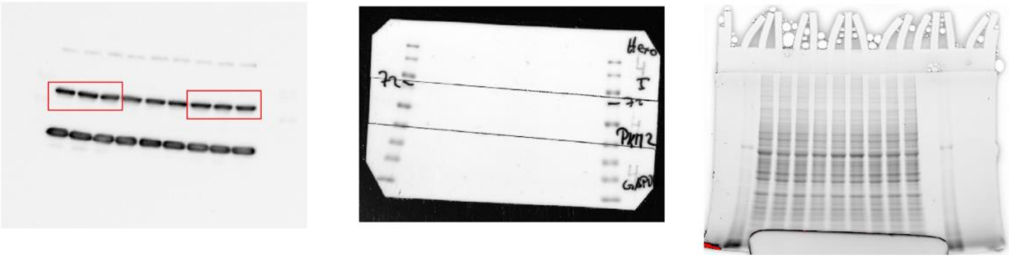

BxPC-3

Pyruvate Dehydrogenase

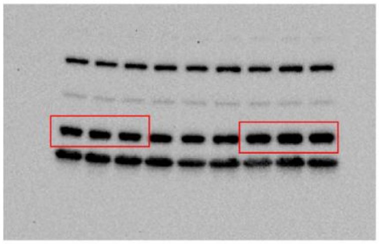

Colorimetric

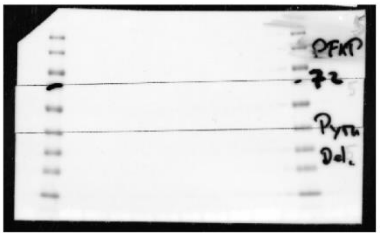

Stain-free loading control

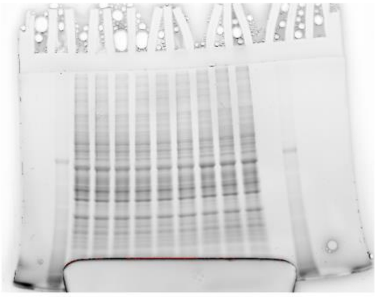

1.7 Figure 3C

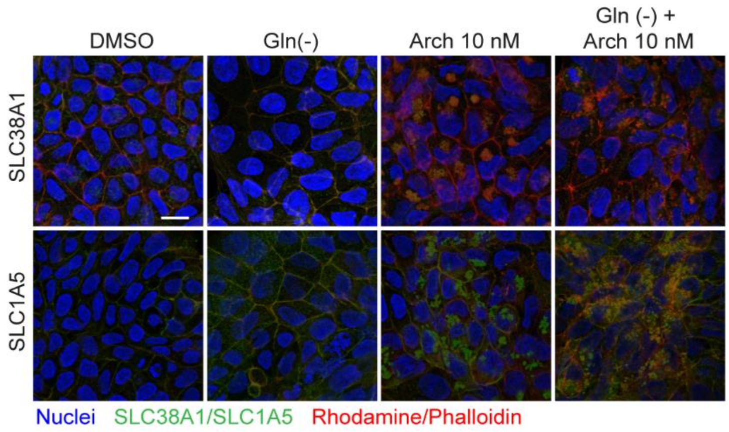
DMSO
Gln(-)
Arch 10 nM
Gln (-) +
Arch 10 nM
SLC38A1
SLC1A5
Nuclei
SLC38A1/SLC1A5
Rhodamine/Phalloidin

2 Supplementary References

1. Robey, R.W., et al., *Inhibition of P-glycoprotein (ABCB1)- and multidrug resistance-associated protein 1 (ABCC1)-mediated transport by the orally administered inhibitor, CBT-1((R))*. Biochem Pharmacol, 2008. **75**(6): p. 1302-12.
